# A Mechanistic Study on the Destabilization of Whole Inactivated Influenza Virus Vaccine in Gastric Environment

**DOI:** 10.1371/journal.pone.0066316

**Published:** 2013-06-11

**Authors:** Hyo-Jick Choi, Charles F. Ebersbacher, Min-Chul Kim, Sang-Moo Kang, Carlo D. Montemagno

**Affiliations:** 1 School of Energy, Environmental, Biological and Medical Engineering University of Cincinnati, Cincinnati, Ohio, United States of America; 2 Center for Inflammation, Immunity and Infection and Department of Biology, Georgia State University, Atlanta, Georgia, United States of America; 3 Emory Vaccine Center and Department of Microbiology and Immunology, Emory University School of Medicine, Atlanta, Georgia, United States of America; 4 National Institute for Nanotechnology, Nanotechnology Accelerator and Department of Chemical and Materials Engineering, University of Alberta, Edmonton, Canada; University of Georgia, United States of America

## Abstract

Oral immunization using whole inactivated influenza virus vaccine promises an efficient vaccination strategy. While oral vaccination was hampered by harsh gastric environment, a systematic understanding about vaccine destabilization mechanisms was not performed. Here, we investigated the separate and combined effects of temperature, retention time, pH, and osmotic stress on the stability of influenza vaccine by monitoring the time-dependent morphological change using stopped-flow light scattering. When exposed to osmotic stress, clustering of vaccine particles was enhanced in an acidic medium (pH 2.0) at ≥25°C. Fluorescence spectroscopic studies showed that hyper-osmotic stress at pH 2.0 and 37°C caused a considerable increase in conformational change of antigenic proteins compared to that in acidic iso-osmotic medium. A structural integrity of membrane was destroyed upon exposure to hyper-osmotic stress, leading to irreversible morphological change, as observed by undulation in stopped-flow light scattering intensity and transmission electron microscopy. Consistent with these analyses, hemagglutination activity decreased more significantly with an increasing magnitude of hyper-osmotic stress than in the presence of the hypo- and iso-osmotic stresses. This study shows that the magnitude and direction of the osmotic gradient has a substantial impact on the stability of orally administrated influenza vaccine.

## Introduction

Severe respiratory disease with substantial morbidity and mortality was associated with influenza viral infection [1]. Continuous evolution of antigenic glycoproteins requires annual reconfiguration of vaccine, specifically matching the new strain, to maintain protective efficacy. As a result of seasonal influenza, an annual average of 250,000 to 500,000 deaths occurred worldwide [2], and 90% of which are elderly persons (≥65 years old) [1]. The annual health costs due to influenza epidemics in the U.S. alone are estimated to be $87.1 billion [3]. The fact that vaccination is the most efficient strategy to prepare for the continuing threat of influenza calls attention to the developments of alternate vaccine, advanced vaccine delivery method, and dose sparing technologies [4].

Currently, the inactivated and live attenuated trivalent vaccines produced in embryonated hen’s egg have been used for intramuscular (IM)/intradermal (ID) and intranasal (IN) administration in the U.S. [5], [6]. The use of hypodermic needles in universal mass vaccination is intrinsically limited due to the need of highly skillful healthcare workers, the risk of biohazard waste, and safety concerns about needle reuse. As a result, there has been extensive research on the development of needle-free vaccine delivery methods and formulations, thereby enabling easier, safer, and cheaper vaccinations in a timelier manner [7]. While injection-based transcutaneous methods are dominantly employed for current vaccination, mucosal immunization has been gaining attention due to advantages including the ability to induce both mucosal and systemic immune responses [8]. Unlike IM injection, which mainly produces virus-neutralizing IgG antibodies, mucosal vaccination can induce the specific immune response of mucosa-associated lymphoid tissue (e.g., secretory IgA antibodies) to prevent pathogen entry into epithelial cells [8]. Among various mucosal vaccination methods, IN administration with the live attenuated influenza vaccine is currently allowed for use in healthy persons aged 5–49 years [5]. However, an inactivated IN vaccine formulation containing adjuvant was withdrawn from the market due to the safety issues such as causing Bell’s palsy [9], [10].

In terms of oral influenza immunization, considerable efforts have been devoted to develop optimal formulations using inactivated virus vaccine due to potential advantages including safety, cost-effectiveness, and ease of production. Because administration through oral route does not require trained personnel, oral vaccine provides a promising platform for the people in developing countries as well as for pandemic influenza preparation. However, *in vivo* animal studies have demonstrated that orally administered influenza vaccine did not produce satisfactory levels of immunogenicity compared with other routes of administration, mainly due to the destabilization of oral vaccines in the stomach [11]–[13]. Thus, to induce a similar level of protective immunogenicity, the vaccine must contain a larger quantity of antigens [8], [11], [14], [15], resulting in the decrease of economic benefits of inactivated oral influenza vaccine. As a result, despite tremendous efforts, there is still no commercially available oral influenza vaccine. Therefore, it is believed that a mechanistic understanding of the effects of the physicochemical gastric environment as a whole on vaccine stability is recognized as a critical step for successful development of oral influenza vaccines.

Ingested vaccine is exposed to low stomach pH at about 37°C during digestion in the stomach. Although gastric digestion rate exhibits a significant level of person-to-person/meal-to-meal variation [16], average transit time for half gastric emptying is estimated to be about 80 min or 127 min for fluid or solid meal, respectively [17]. This indicates that oral vaccines are exposed to gastric juice with a low pH (about 2.0) for about 2 h [18]. Furthermore, enveloped live/inactivated influenza vaccines are susceptible to osmotic stress present in the stomach. Osmotic pressure gradient is generated from osmolarity differences across the lipid membrane, which has been known to influence structural/functional integrity of organisms [19]–[21]. Thus, osmotic pressure-induced functional activity loss of vaccine has been recognized as a major obstacle to the development of vaccines [22]. Considering oral vaccines are subjected to these harsh conditions, systematic studies on time-dependent vaccine stability change at low pH and physiological temperature in the presence of osmotic stress are essential to understand vaccine destabilization mechanisms. Since pH-induced antigen denaturation has been previously reported [23], [24], the same principle has been exclusively adopted to explain lower level of immunogenicity of orally administered influenza vaccine. Interestingly, stability of oral vaccine has been studied with over-simplified conditions and a time-dependent detailed analysis has not been carried out so far under conditions similar to gastric environments. Thus, other major gastric conditions such as temperature, osmotic stress, and gastric residence time were neglected in the analysis. It is unlikely that only pH-induced irreversible conformational change of hemagglutinin (HA) contributes to activity loss of oral vaccine during the whole gastric digestion process.

Therefore, we performed experiments using stopped-flow light scattering (SFLS) analysis to observe time-dependent, osmotic stress-induced morphological change of the whole inactivated influenza virus vaccine under physiological gastric conditions. The underlying hypothesis is that low pH-induced conformational change of HA protein leads to a decrease of vaccine efficacy at the initial stage, while osmotic stress, combined with other gastric environmental factors (temperature, osmotic stress, dilution effect, and retention time) results in further decrease of vaccine efficacy. SFLS is commonly used to examine reactions kinetics after small volumes of solutions are mixed. For this purpose, a correlation between osmotic swelling/shrinking behavior and scattered light intensity change was established for liposomes made of phosphatidylcholine, i.e. one of major lipid components of the egg-grown viruses, as a model system for influenza vaccine [25], [26]. This relationship was then used in quantitative analysis of morphological variation in terms of influenza vaccine size change at different test conditions. Transmission electron microscopy (TEM), intrinsic fluorescence, and hemagglutination activity were subsequently measured to determine how functional activity of HA can be related to the time-dependent membrane perturbation of the virus.

## Materials and Methods

### Preparation of Influenza Vaccine

Inactivated A/PR/8/34 (H1N1) influenza A virus was used as influenza vaccine, and prepared as described previously [27]. Briefly, A/PR/8/34 virus was grown in chicken eggs for 2 days at 37°C. Allantoic fluids of infected eggs were harvested and centrifuged to remove cell debris. The virus was purified by ultracentrifugation (at 28,000 rpm for 60 min). The purified virus was inactivated with formalin at a final concentration of 1∶4,000 (v/v).

### Preparation of Liposomes

Liposomes were prepared by the thin film hydration method, followed by extrusion [28]. Briefly, chloroform solution of egg phosphatidylcholine (egg PC, 100 mg/ml; Avanti polar lipids, Alabaster, AL) was rotary evaporated under vacuum in a round-bottom flask at 40°C and the resulting lipid film was hydrated with 600 mM sucrose solution (PBS, pH 7.0) or PBS (pH 7.0) giving a final PC concentration of 10 mg/ml at 4°C for 2 h. Then, the liposomes (average radius: 84.2±4.8 nm) were extruded through 220 nm pore membranes 12 times at room temperature (R.T.).

### Dynamic Light Scattering Analysis of Liposomes

The osmotic stress-induced change in liposome size was characterized by dynamic light scattering (DLS) (Zetasizer Nano S; Malvern Instruments USA, Westborough, MA). DLS measurements were performed at 20°C with the scattering angle at 173° after 1∶1 mixing of liposome with sucrose solutions. Liposome suspensions in 600 mM sucrose (PBS, pH 7.0) were mixed with hypo-osmotic sucrose solutions (0, 100, 200, and 300 mM sucrose in PBS, pH 7.0) to observe hypo-osmotic stress-induced size change of liposomes. Liposomes suspended in PBS were mixed with iso-osmotic (PBS, pH 7.0) and hyper-osmotic (200, 300, 500, 600, 800, 1000, 1250, and 1500 mM sucrose in PBS, pH 7.0) sucrose solutions to measure the size change of liposomes in iso- and hyper-osmotic stress conditions, respectively. The viscosity of the sucrose solution was measured at 20°C using glass capillary viscometers (Cannon Instrument, State College, PA). Refractive index and dielectric constant were calculated from the reported data [29], [30].

### Stopped-flow Light Scattering Analysis

Osmotic stress-induced swelling/shrinking behavior of liposome/vaccine was investigated by using a stopped-flow apparatus (MOS-200/M spectrometer, SFM-20; Bio-Logic USA, Knoxville, TN). The light scattering intensity was recorded as a function of time immediately after the mixing of the liposome/vaccine samples with various concentrations of sucrose in an equal volume ratio (injection volume: 66 µl, flow rate: 7 ml/s). The sample temperature was controlled at five different temperatures (4, 15, 25, 31, and 37°C) with a recirculating water bath. Emission spectra were recorded with an excitation wavelength of 546 nm. Rate constant (*k*) was obtained from the curve fitting of the light scattering spectra using Bio-Kine 32 V4.46 software (Bio-Logic).

In this work, sucrose was used as an osmolyte, and osmolarity (1 osM = 1000 mosM (milliosmolarity)) was calculated from the final sucrose concentration (C), assuming ideal solution. The osmotic gradient across the liposome membrane/viral membrane was defined as the difference in osmolarity between the external and inner medium (Δ = C_ex_ – C_in_, expressed as osM or mosM, depending on convenience to better represent the data). The osmolarity of the inner viral compartment was measured to be about 300 mosM via measurements of its equilibrium osmolarity with outer medium using stopped-flow spectrophotometer.

SFLS measurements with liposomes were performed at 4°C in response to the same osmotic pressure as in DLS analysis; i.e., hypo-osmotic (Δ = −0.3, −0.25, −0.2, and −0.15 osM), iso-osmotic (Δ = 0 osM), and hyper-osmotic (Δ = 0.1, 0.15, 0.225, 0.3, 0.4, 0.5, 0.625, and 0.75 osM) gradients. Stability of vaccine in simulated gastric environment was investigated by analyzing scattered light intensity change at pH 2.0 and 37°C under various osmotic pressures (Δ = −150, 0, 150, and 300 mosM). The scattered light was recorded right after mixing vaccine stock solution (300 mM sucrose, pH 7.0) with osmolyte solutions (0, 300, 600, and 900 mM sucrose solution at pH 1.7) at 37°C. To compare the effect of pH on vaccine stability, SFLS analysis was performed using osmolyte solution at pH 7.0 with all other conditions the same as before. Light scattering was mainly measured over a 2-h incubation period and additional scanning (scan time: 4 s and 100 s) was performed to better characterize morphology at a very early stage of incubation.

### Electron Microscopy

The morphology of influenza vaccine was analyzed using TEM (Philips/FEI CM20; FEI, Hillsboro, OR). In case of incubation in osmotic solution at pH 7.0/2.0, vaccine samples were centrifuged (12,000 rpm, 20 min) after incubation for 2 h and resuspended in PBS solution (pH 7.0). To prepare TEM samples, a drop of vaccine suspension (1 mg/ml) was placed on a 3 mm formvar-amorphous carbon-coated copper grid (Ted Pella, Redding, CA). After 7 min, excess solution was blotted off using filter paper and samples were then stained with 2% phosphotungstic acid (pH = 7.0; Electron Microscopy Sciences, Hatfield, PA). TEM was operated at 200 kV.

### Effects of Osmotic Stress on the Functional HA Activity of Influenza Vaccine at Low pH

To investigate the effects of osmotic gradients on vaccine at pH 2.0 as a function of incubation time, functional HA activity of the inactivated virus was tested using hemagglutination assay. Vaccine in iso-osmotic conditions (vaccine suspension in 300 mM sucrose solution, pH 7.0) was rapidly added to the preheated sucrose solutions (1 mg/ml in 0, 300, 600, 900, and 1300 mM sucrose solution at pH 1.7, 37°C) in an equal volume ratio to impose the desired hypo-, iso-, or hyper-osmotic stress on vaccine (Δ = −150, 0, 150, 300, and 500 mosM). The vaccine-acid mixture was incubated at 37°C in an oven and aliquot was taken at various times (1, 5, 15, 30, 60, 90, and 120 min) for HA activity measurement. This 2 h incubation was performed in sealed Eppendorf tubes to prevent evaporation of aqueous solution.

For the 1 µl aliquot taken at each time point, the sample was diluted in 99 µl of PBS and further serially diluted in PBS before adding a 0.7% suspension of chicken red blood cells (Lampire Biological Laboratories, Pipersville, PA). All HA titer experiments were performed at 4°C to eliminate the temperature effect on the remaining HA activity. HA activity of vaccine in iso-osmotic condition at 4°C (pH 7.0) was taken as a reference and HA activity of the samples at each osmotic condition was calculated relative to it.

### Fluorescence Spectroscopy

The conformational stability of antigenic proteins of vaccine was investigated by measuring intrinsic fluorescence with fluorescence spectroscopy [31]. Fluorescence was measured with a vaccine concentration of 80 µg/ml at the excitation wavelength of 295 nm over the scanning range of 310–420 nm (slit size: 10 nm, scanning speed: 200 nm/min) in quartz cuvette (Stama Cells, Atascadero, CA) using a fluorimeter (LB 50B; PerkinElmer, Waltham, MA).

Inactivated influenza virus suspension in 300 mM sucrose (pH 7.0) and osmolyte sucrose solution (300 mM (iso-osmotic) and 900 mM (hyper-osmotic), pH 1.7) were preheated to 37°C, and mixed to investigate structural stability of vaccine at pH 2.0 over the increase of incubation time in a preheated oven at 37°C. The acquired fluorescence spectra were corrected for background signal by measuring the same solution in the absence of vaccine. As a reference, fluorescence emission spectra were measured at zero time under iso-osmotic condition after mixing with 300 mM sucrose solution (pH 7.0) at both 4°C and 37°C. The vaccine-acid samples were taken at 1, 5, 15, 30, 60, 90, and 120 min for intrinsic fluorescence measurement. Then, the peak shift and relative intensity change of emission maxima compared with those of a reference were used to evaluate relative stability of vaccine. The effect of osmotic stress increase on the stability of vaccine was examined by recording intrinsic fluorescence in the presence of osmotic gradients (0, 50, 100, 150, 200, 300, 400, 500, 600, 750, and 1000 mosM) after incubation for 2 h at pH 7.0 and 37°C. After appropriate background correction, fluorescence spectra were examined with reference to the spectrum of vaccine in iso-osmotic condition at pH 7.0.

### Effect of the Dilution on the Functional HA Activity of Vaccine

To examine the effect of dilution on the functional HA activity, vaccine stock solution of 5 mg/ml and 25 mg/ml of vaccine stock solution in 300 mM sucrose solution (pH 7.0, 37°C) was diluted to 10-fold and 50-fold, respectively. Diluted vaccines were then mixed with osmolyte solutions (300, 900, and 1300 mM sucrose solution, at pH 7.0 and 37°C) to impose iso-osmotic and hyper-osmotic (Δ = 300 and 500 mosM) stress to vaccine and incubated in an oven at 37°C for 2 h. From the vaccine-sucrose mixture, 2 µl aliquot was diluted in 98 µl of PBS for HA titer measurement. HA activity of vaccine was calculated relative to the reference sample (HA titer of 1 µg of vaccine in iso-osmotic condition, at pH 7.0 and 4°C).

### Statistics

Student’s *t*-test or analysis of variance (ANOVA; one-way or two-way ANOVA with Tukey’s post hoc test) (Minitab release 14; Minitab, State College, PA) was used to analyze data. A *P* value of less than 0.05 indicated a significant difference.

## Results

### Relationship between Scattered Light Intensity and Liposome Size

The change in the size of vaccine was calculated using the experimentally determined correlation between the SFLS intensity and the size of reference PC-liposomes. PC-liposomes function as a reference because their size can be determined from DLS while the equivalent SFLS spectrum from the same liposomes can be obtained. Knowing the exact volume and the corresponding SFLS spectrum allows for a relationship to be established that can be applied to influenza vaccines composed of similar phospholipids.

#### 1. Stopped-flow light scattering analysis of PC-liposomes

**Figure 1:**
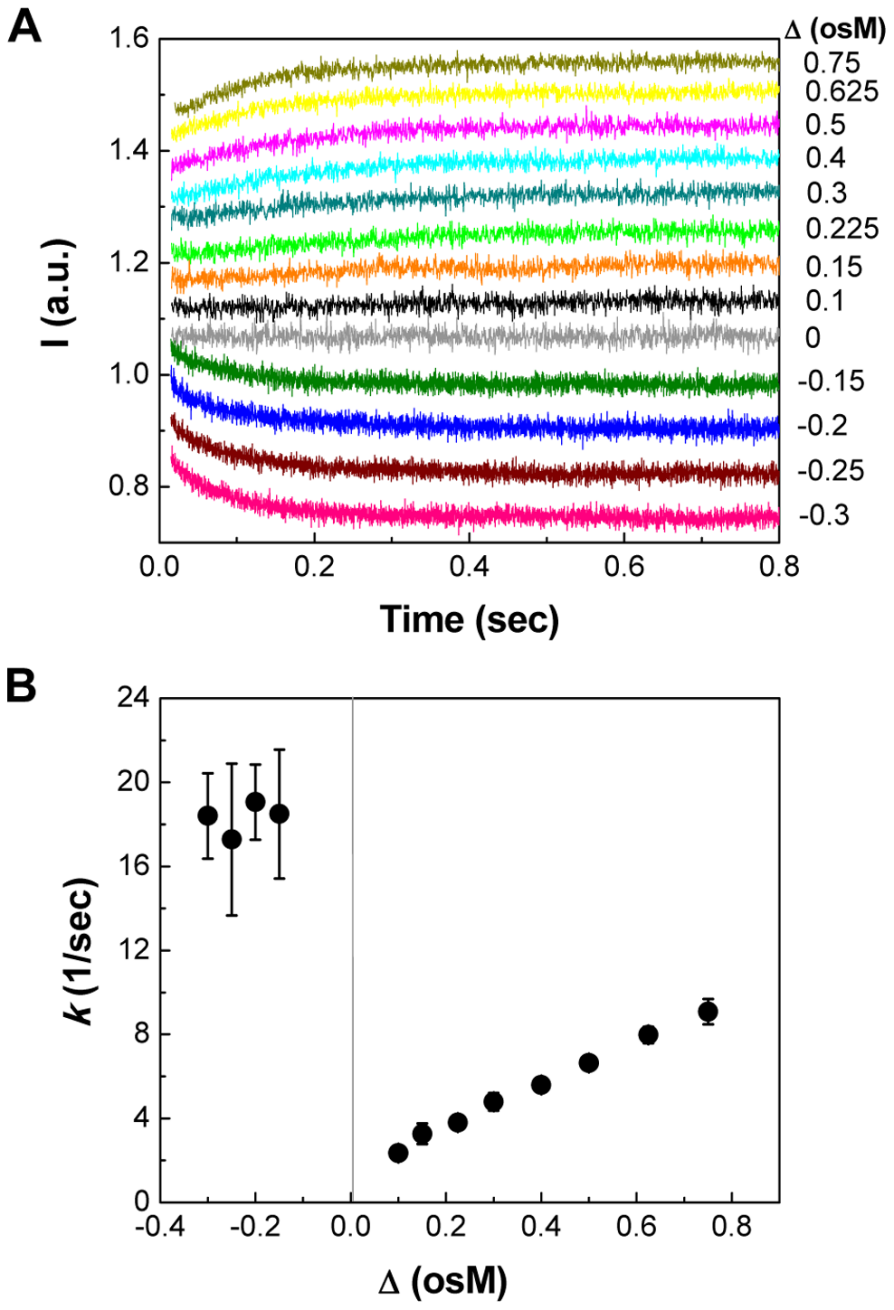
Stopped-flow light scattering (SFLS) measurement of osmotic behavior of liposomes. (A) Time course of SFLS analysis of phosphatidylcholine (PC)-liposomes. Liposome suspension was exposed to osmolyte solutions containing different concentrations of sucrose in a SFLS apparatus and the resulting changes in scattered light intensity (I) were recorded at 546 nm for 0.8 s. Osmotic stress (Δ = C_ex_ – C_in_) was controlled by changing osmolarity (osM = 1000×milliosmolarity (mosM)) of both internal (C_in_) and external (C_ex_) medium of liposomes. (B) Rate constant (*k* [1/s]) as a function of osmotic stress. The relative light scattering data (I_rel_ = I/I_0_, I_0_: initial intensity at time zero, I: intensity at time t) were curve-fitted using the equation I = a+b·e^–*k*·t^ where a and b are constants and *k* is a rate constant, and corresponding rate constants were presented as the mean ± standard deviation (SD) (*n* = 54). Hyper-osmotic (Δ>0) shrinkage and hypo-osmotic (Δ<0) swelling of liposomes result in an increase and a decrease of light scattering intensity, respectively.

Figure 1A shows SFLS curves observed from liposomes as a function of osmotic stress (Δ). The time course of the change in scattered light intensity (I) gave a typical osmotic behavior, depending on the direction and magnitude of osmotic pressure. As shown in the plot, iso-osmotic stress (Δ = 0) did not show any significant time-dependent change in scattering intensity and thus no size change occurred. On the contrary, SFLS intensity decreased with incubation time due to the swelling of liposomes in response to hypo-osmotic stress (Δ<0), and increased by the shrinkage of liposomes in hyper-osmotic medium (Δ>0).

The rate constant (*k*), a parameter describing the size change rate, is plotted as a function of osmotic gradient (Figure 1B). *k* values were significantly higher in the case of hypo-osmotic stress (*k* ∼ 18, Δ = −0.3 osM) as compared with hyper-osmotic stress (*k* ∼ 5, Δ = 0.3 osM) (two-way ANOVA, *P*<0.005). This means that the resistance of liposomes to shrinkage is higher than to swelling, which is consistent with a previous report [32].

#### 2. Relation between I_rel_ and V_rel_ of PC-liposomes

In order to establish a relationship between SFLS intensity and liposome volume, it is required to express both relative intensity (I_rel_ = I_sat_/I_0_, I_0_: initial intensity at 0 s, I_sat_: saturation intensity) of scattered light and relative volume (V_rel_ = V_Δ_/V_iso_, V_iso_: volume in iso-osmotic solution, V_Δ_: volume in hypo- or hyper-osmotic stress) of liposomes in terms of osmotic stress.

The time course of I_rel_ for liposomes is shown in Figure S1 as a function of inverse osmolarity. I_rel_ at each osmotic gradient was calculated by dividing the saturation scattering intensity (I_sat_ = a, as t → ∞) by the initial scattering intensity (I_0_ = a+b, at t = 0), i.e. a/(a+b), using the results obtained from curve-fitting analysis of SFLS curves shown in Figure 1A. As shown in Figure S1, I_rel_ data points were well fitted to an empirical function I_rel_ = 1+ c·e^x/Δ^+d·e^y/Δ^, where c, d, x, and y were −0.11653, −2.28333, 0.09645, and 1.61951, respectively, for hypo-osmotic stress and 0.14824, 5.14043, −0.21065, and −3.30994, respectively, for hyper-osmotic stress to establish the relationship between I_rel_ and Δ.

**Figure 2:**
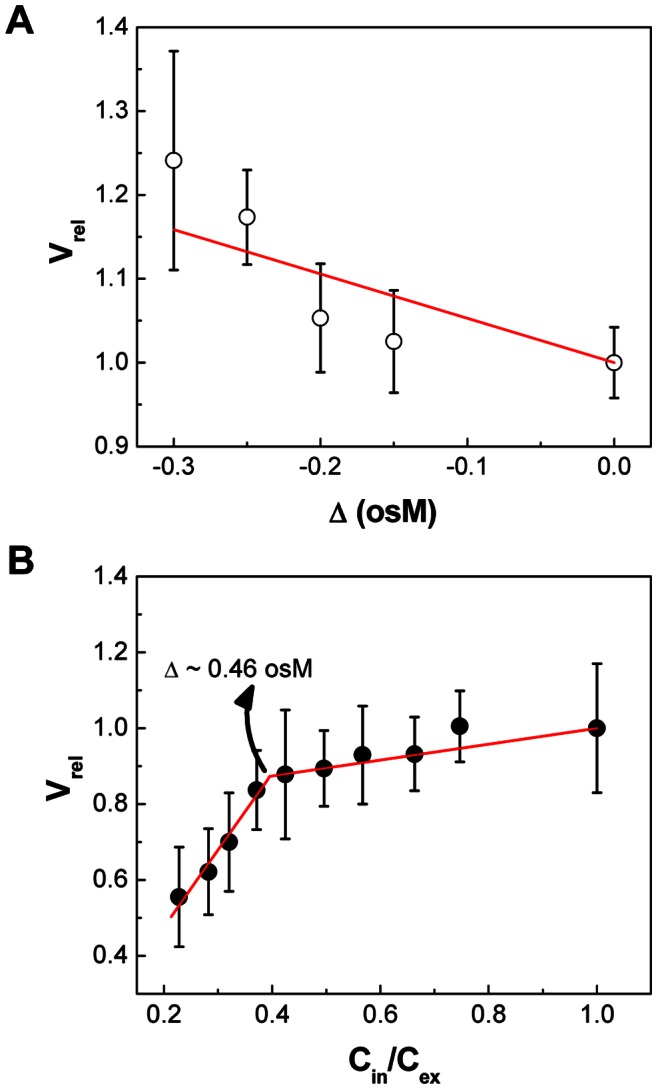
Osmotic stress dependence of the relative volume (V_rel_) of PC-liposomes. V_rel_ of liposomes at (A) hypo-osmotic and (B) hyper-osmotic conditions. (Mean ± SD; *n* = 36–60.).

The PC-liposome volume change relative to the iso-osmotic volume was determined using DLS analysis, as shown in Figures 2A and 2B for hypo-osmotic and hyper-osmotic conditions, respectively. Figure 2A shows that V_rel_ increases due to swelling upon exposure to hypo-osmotic stress. The data were fitted by a linear regression analysis: V_rel_ = 1–0.52879·Δ. When submitted to hyper-osmotic stress (Figure 2B), V_rel_ of liposomes exhibited a linear shrinkage behavior with two distinct phases; i.e., V_rel_ = 0.79+0.21·(C_in_/C_ex_) for 0< Δ ≤0.46 and V_rel_ = 0.07+2.03·(C_in_/C_ex_) for Δ ≥0.46. Therefore, the dependence of V_rel_ on I_rel_ can be derived using a common variable Δ ( = C_ex_ – C_in_) to both equations. That is, the relative scattering intensity of SFLS curve (I_rel_(t) = I(t)/I(0)) can be converted into the corresponding osmotic stress at each time point, which can then be used to find time-dependent relative volume change of liposomes (V_rel_(t) = V(t)/V(0)).

### Effects of pH and Temperature on the Hyper-osmotic behavior of Influenza Vaccine

The influence of pH and temperature was estimated by analyzing SFLS curves obtained from influenza vaccine (87±17 nm in diameter from TEM image analysis). For precise measurements, the change in scattered light intensity was recorded using different scan ranges. Rapid morphological change from initial osmotic behavior was monitored by SFLS for 8 s and longer-term morphological change was separately monitored by scanning for 160 s.

#### 1. Effect of temperature on the I_rel_ – V_rel_ of influenza vaccine at pH 7.0

**Figure 3:**
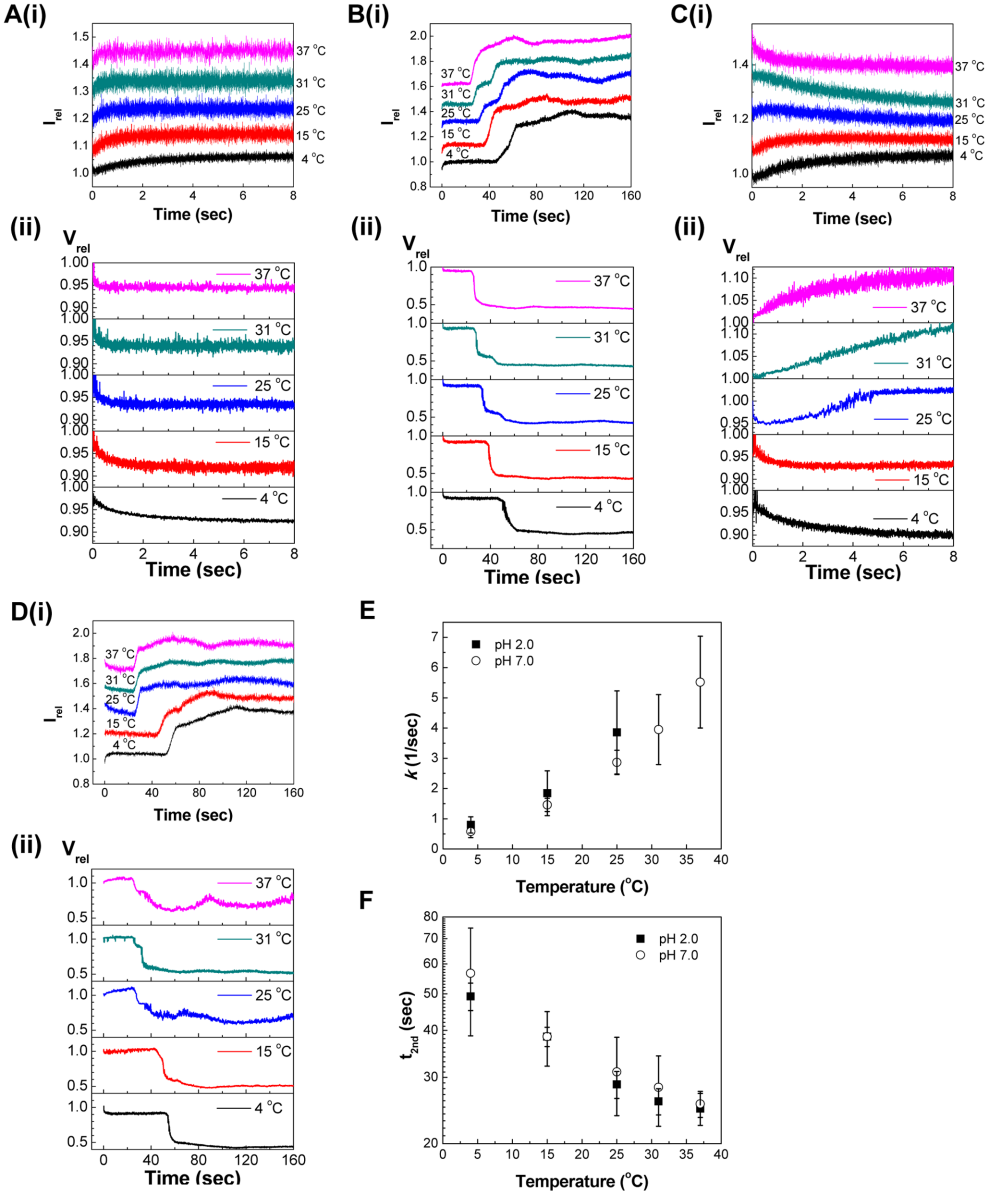
The effects of temperature and pH on the time-dependent osmotic response of inactivated A/PR/8/34 influenza virus vaccine. The osmotic shrinkage behavior of the virus was investigated at five different temperatures (4, 15, 25, 31, and 37°C) by applying a hyper-osmotic gradient of 300 milliosmolarity (i.e., Δ = 300 mosM or 0.3 osM) with different pH sucrose solutions (pH 2.0 and 7.0). 8-s (A, C) and 160-s (B, D) scan of SFLS of influenza vaccine in response to sucrose solution at pH 7.0. (A, B) and 2.0 (C, D). (i) I_rel_ of SFLS spectra are offset to highlight the differences, but the relative intensity scale is identical for all spectra. (ii) V_rel_ of influenza vaccine corresponding to SFLS in part (i). Osmotic gradient (Δ) corresponding to I_rel_ at each time point was calculated from equations in Figure S1 using Wolfram|Alpha (www.wolframalpha.com), and V_rel_ was calculated using Δ from equations shown in Figures 2A and 2B. (E) Rate constant (*k*) at the primary shrinkage phase as a function of temperature (Mean ± SD; *n* = 96). (F) Onset time for the secondary shrinkage (t_2nd_) as a function of temperature (Mean ± SD; *n* = 42). Data (A(i) and C(i)) were curve-fitted to the equation in Figure 1B.

SFLS analysis was performed on the influenza vaccine after exposure to hyper-osmotic solution (Δ = 300 mosM, pH 7.0) at 4, 15, 25, 31, and 37°C. The light scattering was recorded for 8 s and the resulting I_rel_ and corresponding V_rel_ were represented in Figures 3A(i) and 3A(ii), respectively. I_rel_ was converted to V_rel_ using the relationship described above. As shown in Figure 3A(i), influenza vaccine exhibited an increase in I_rel_ due to hyper-osmotic shrinkage, consistent with the observation from liposomes. It was also interesting to note that I_rel_ reached saturation earlier with increasing temperatures, which translates to faster shrinkage. From the V_rel_-time plot, influenza vaccine was found to shrink to about 92–95% of original volume after incubation for 8 s (Figure 3A(ii)). The trend in the time-dependent V_rel_ change in Figure 3A(ii) showed a good correlation to the scattering intensity curves in Figure 3A(i).

Longer-term morphological change of the influenza vaccine was investigated by analyzing SFLS spectra monitored for 160 s. Under the same testing conditions as in Figure 3A, SFLS exhibited a step-wise intensity increase (Figure 3B(i)), corresponding to about 90% and 45% of the original volume during the first and secondary shrinkage stages, respectively (Figure 3B(ii)). Furthermore, the onset time for the secondary shrinkage (t_2nd_) decreased with increasing temperature. Based on the previous prediction about the step-wise shrinkage of influenza vaccine, this may be explained by the lowered resistance of matrix proteins (M1) to further shrinkage due to increased thermal energy [33].

#### 2. Effect of temperature on the I_rel_ – V_rel_ of influenza vaccine at pH 2.0

Time-dependent morphological change of influenza vaccine at low pH (2.0) was investigated under the same test conditions as pH 7.0. Figure 3C shows the time-course of I_rel_ (i) and V_rel_ (ii) measured for 8 s at various temperatures. Influenza vaccine at 4 and 15°C exhibited a typical hyper-osmotic shrinkage behavior as evidenced by increased I_rel_ (Figure 3C(i)) indicating V_rel_ decrease (Figure 3C(ii)). However, I_rel_ began to decrease at ∼ 0.6 s (25°C) after an initial increase. As shown in Figure 3C(ii), the initial increase and subsequent decrease in I_rel_ can be understood in terms of V_rel_ change: influenza vaccine shrank to 95% for up to ∼ 0.6 s upon exposure to hyper-osmotic stress (300 mosM), followed by an increase to 102% at 8 s. Previous report about clustering of ectodomain-bound liposomes at low pH in an intermolecular fashion indicates such a reversal in the direction of vaccine size change can be attributed to vaccine aggregation by acid treatment [34]. Thus, although influenza vaccine was subjected to hyper-osmotic shock, no evidence of hyper-osmotic shrinkage was observed at pH 2.0 and 37°C (V_rel_ = 110% at 8 s) with short-term observations.

Longer-term course of SFLS analysis was performed on the same samples for 160 s (Figure 3D). Under hyper-osmotic stress, a step-wise shrinkage was observed at 4 and 15°C (pH 2.0), similar to pH 7.0 (compare Figure 3D with Figure 3B). At 4°C, the primary and secondary shrinkage phase resulted in 92% and 42% of the original size, respectively. Also, as predicted from the short-term SFLS data (Figure 3C), a decrease in I_rel_ was clearly observed at the first phase in SFLS curves at 25, 31, and 37°C (Figure 3D(i)). However, the trend for increasing size at the first phase started to decrease at ∼ 24 s, as demonstrated by the I_rel_ increase (Figure 3D(i)) and gradual V_rel_ decrease (Figure 3D(ii)). This analysis indicates that while the average virus size increased by aggregation in response to acidic hyper-osmotic solution at high temperatures (25, 31, and 37°C), a longer incubation resulted in typical hyper-osmotic volume changes. Thus, at 37°C, V_rel_ of influenza vaccine increased to 108% at the first phase followed by a decrease to a minimum of 60% (Figure 3D(ii)). It is also noted that at temperatures higher than 25°C, the secondary volumetric change at pH 2.0 initiated after similar time lags to pH 7.0, but proceeded more slowly than at pH 7.0 (compare Figure 3D(ii) with 3B(ii)). This can be explained by the aggregation-induced physical hindrance effect, thereby resisting osmotic shrinkage, and/or by the decreased osmotic gradient across the vaccine membrane due to the low pH-induced membrane leakage, as evidenced by lower levels of shrinkage compared to that at pH 7.0. Furthermore, noticeably more fluctuation in V_rel_ at ≥25°C showed that high temperature are more detrimental to the physical stability of the enveloped influenza vaccine under hyper-osmotic stress at pH 2.0 (Figure 3D(ii)), consistent with previous reports [35].

#### 3. Effects of pH (2.0 and 7.0) and temperature on the shrinkage kinetics of influenza vaccine

The effects of pH on the shrinkage kinetics were studied by analyzing SFLS curves in terms of *k* during the primary shrinkage and t_2nd_. Figures 3E and 3F show that *k* and t_2nd_ values from Figures 3A(i) and 3C(i) varied significantly depending on temperature (two-way ANOVA, *P*<0.005). As discussed in Figure 3C, the lack of an intensity increase at the primary stage prevented measurement of the initial hyper-osmotic shrinkage rate constant at pH 2.0 and ≥25°C (Figure 3E). *k* values for both pH 7.0 and 2.0 increased with temperature (Figure 3E), but varied with a significant statistical difference between the two pH conditions (two-way ANOVA, *P*<0.001). On the other hand, while a significant decrease in t_2nd_ was seen with increasing temperatures (Figure 3F), the results exhibited no statistically significant effects of pH on t_2nd_ (two-way ANOVA, *P* = 0.143). This analysis provides a basis for further understanding of the initial vaccine size increase under hyper-osmotic stress at pH 2.0 (Figures 3C and 3D). If the vaccine size increase is caused by fusion, the osmolyte leakage through membrane defects during the fusion process may reduce the osmotic gradient across the lipid membrane. Since t_2nd_ is associated with the magnitude of osmotic gradient [33], such a lowered osmotic gradient would extend t_2nd_. In addition, vaccine fusion with an accompanying size increase would lead to the smaller *k* values for the initial shrinkage process. Therefore, based on the observations of (1) the statistically higher *k* values during the first shrinkage step at pH 2.0 than those at pH 7.0 with increasing temperature up to 25°C (Figure 3E) and (2) the same t_2nd_ (Figure 3F) at both pH 7.0 and 2.0, the temporary increase in V_rel_ at pH 2.0 in the presence of hyper-osmotic stress is further supported to be related to aggregation, rather than fusion. Although further research is needed to understand the mechanisms of *k* increase at pH 2.0 compared to pH 7.0, these SFLS results clearly show that exposure of vaccine to acidic environments reduced the resistance to the primary shrinkage, without any significant membrane integrity loss.

### Effects of Osmotic Stress and pH on the Stability of Influenza Vaccine at 37°C

#### 1. Osmotic response of vaccine

**Figure 4:**
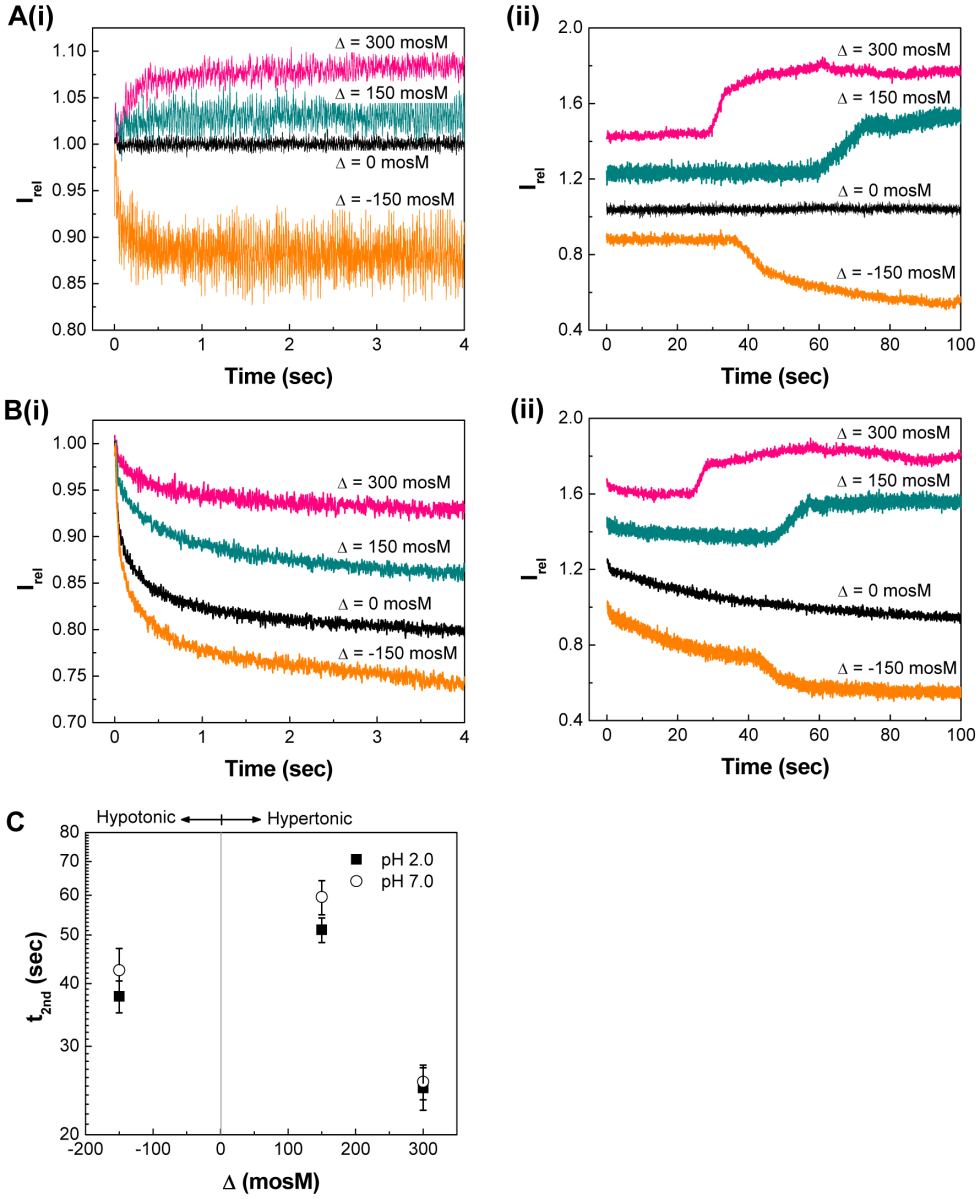
A comparison of pH-dependent osmotic swelling/shrinking behavior of influenza vaccine. SFLS analysis of the virus subjected to osmotic gradient of −150, 0, 150, and 300 mosM using sucrose at (A) pH 7.0 and (B) pH 2.0 at 37°C. Scan time of (i) 4 s and (ii) 100 s. 100-s scan spectra are offset for clarity. (C) t_2nd_ as a function of osmotic gradient (−150, 150, and 300 mosM) at pH 7.0 and 2.0. (Mean ± SD; *n* = 24–36.).

SFLS analysis was performed by applying four different osmotic stresses (Δ = −150, 0, 150, and 300 mosM) to influenza vaccine at pH 2.0/7.0 and 37°C (see Figures 4A/4B for pH 7.0/2.0 and (i)/(ii) for 4−/100-s scan). As shown in Figure 4A(i), SFLS spectra at pH 7.0 displayed a typical osmotic response, similar to the liposomes (Figure 1). That is, SFLS intensity decreased upon exposure to Δ = −150 mosM due to swelling and increased due to hyper-osmotic shrinkage at Δ = 150 and 300 mosM. In the iso-osmotic condition, no significant level of time-dependent intensity variation was observed. As shown in Figure 4A(ii), influenza vaccine shrank in a step-wise manner after exposure to hyper-osmotic stress, as described in Figure 3. Furthermore, the decrease of t_2nd_ with increasing hyper-osmotic stress was consistent with a previous report [33]. Another important observation is that swelling of influenza vaccine also proceeded in a similar manner to hyper-osmotic shrinkage, as indicated by the step-wise intensity decrease at Δ = −150 mosM (Figure 4B(ii)).

At pH 2.0, SFLS spectra exhibited a decrease in I_rel_ at the initial stage of incubation for all tested osmotic stresses, as shown in Figure 4B(i). This confirms that low pH-induced vaccine aggregation is a general phenomenon that occurs at pH 2.0 and 37°C. Such a gradual decrease in I_rel_ was also observed throughout the whole incubation period at the iso-osmotic stress (Figure 4B(ii)). From the analysis of 100-s scans, t_2nd_ was found to vary significantly for Δ = −150 and 150 mosM (two-way ANOVA, *P*<0.005), but not for Δ = 300 mosM (one-way ANOVA, *P* = 0.527). While not fully understood, it is hypothesized that acidic pH-exposed influenza vaccine could be subject to a moderate decrease in the resistance of M1 layer, resulting in a slight enhancement of the secondary swelling/shrinking process at relatively low osmotic stress (Δ = −150 and 150 mosM) at pH 2.0, but not big enough to observe a significant difference at higher osmotic stress (Δ = 300 mosM). It is also clear that influenza vaccine exhibits less resistance to swelling than to shrinkage by looking at the smaller t_2nd_ at −150 mosM than at 150 mosM, consistent with cells [36].

**Figure 5:**
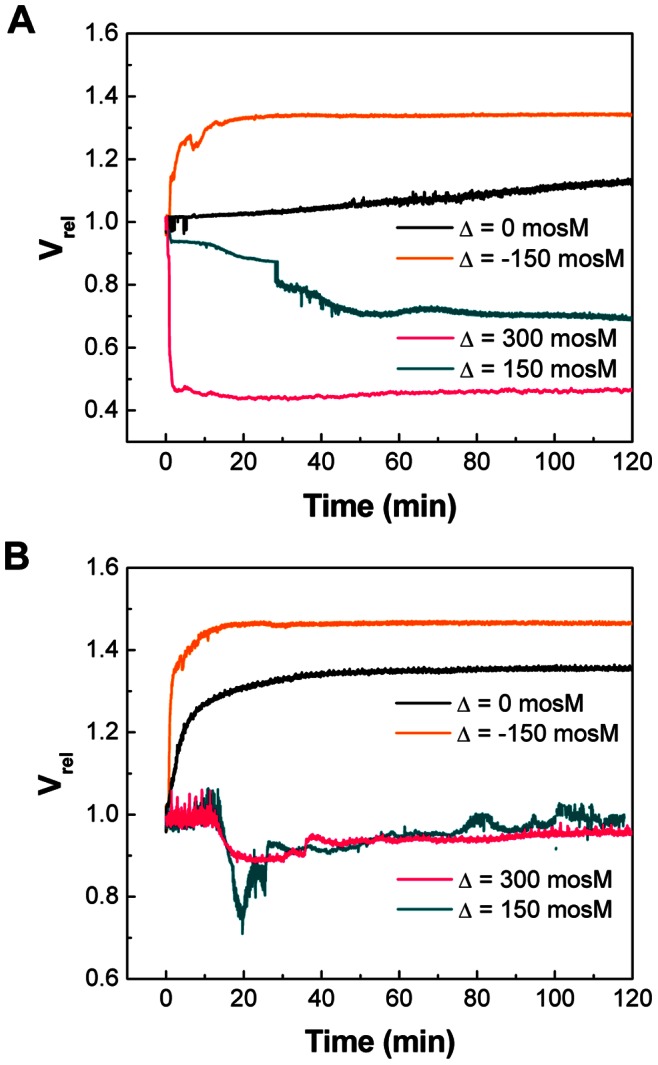
Effects of pH and osmotic stress on the morphological change of influenza vaccine. The V_rel_ changes of vaccine subjected to osmotic stress of −150, 0, 150, and 300 mosM at pH 7.0 (A) and 2.0 (B) at 37°C for 2 h. The corresponding SFLS curves of pH 7.0 and 2.0 are shown in Figures S2 and S3A, respectively. Spectra are representative of *n* = 9 replicate samples examined at each condition.

Time-course of SFLS analysis was carried out for 2 h to simulate general osmotic response of influenza vaccine during the gastric digestion process. SFLS curves at pH 7.0 (Figure S2) were measured for comparison with those at pH 2.0 (Figure S3) to estimate the pH effects at 37°C. Influenza vaccine showed the expected swell-shrink characteristics against hypo- and hyper-osmotic stress at pH 7.0 (Figure S2 for I_rel_ and 5A for V_rel_). As shown in Figure 5A, V_rel_ decreased to ∼ 44% at Δ = 300 mosM and no significant size variation was observed with further incubation up to 2 h at pH 7.0, which is consistent with the findings in Figure 3B. In the case of Δ = 150 mosM, a less hyper-osmotic shrinkage (V_rel_ = ∼ 70%) was observed compared to that from Δ = 300 mosM as a consequence of lower osmotic stress. When exposed to Δ = −150 mosM, V_rel_ increased to ∼ 134% and the hypotonically swollen vaccine was stably maintained up to 2 h without disruption. It was also noted that influenza vaccine in iso-osmotic stress generated a gradual decrease in I_rel_ (Figure S2) and thereby a slow increase in size, resulting in V_rel_ = ∼ 113% after 2 h incubation (Figure 5A). It is estimated that such a size increase in iso-osmotic condition may be associated with vaccine destabilization due to dilution, as had been reported earlier for liposomes [37], [38]. Taking into account of 13% volume increase with dilution, the osmotic stress-induced net volume was calculated to be ∼ 121% (Δ = −150 mosM), ∼ 57% (Δ = 150 mosM), and ∼ 31% (Δ = 300 mosM), relative to that of virus in iso-osmotic condition (i.e., 100%), after incubation for 2 h at pH 7.0 and 37°C.

Figures S3 and 5B show I_rel_ (i) and V_rel_ (ii) at pH 2.0. As shown in Figure 5B, V_rel_ increased and stably remained at 146% at Δ = −150 mosM after 2 h incubation. The previously mentioned V_rel_ increase from dilution at iso-osmotic condition was more dominantly observed at pH 2.0, resulting in V_rel_ ∼ 136%. Considering the contribution of the dilution effect, the additional 23% increase in V_rel_ can be explained by the previously mentioned low pH-induced aggregation. Thus, the net V_rel_ change due to 150 mosM of hypo-osmotic stress is analyzed to have further increased another 10% compared to influenza vaccine at Δ = 0 at pH 2.0. By contrast, the influenza vaccine exhibited very unstable light scattering patterns at hyper-osmotic stress (see Figures S3B and S3C for magnified SFLS spectra and V_rel_ corresponding to Δ = 150 and 300 mosM, respectively). Thus, similarly to the explanation shown in Figure 3D, it is predicted that membrane defects formed during deformation potentially functioned as a leakage path for osmolyte diffusion, causing the osmotic gradient to decrease. The fact that irregular scattering was accompanied by a subsequent membrane deformation can be supported by the observation of no appreciable change in V_rel_ after 2 h incubation (98% for 150 mosM and 96% for 300 mosM) as a result of dissipation of osmotic gradient. Considering that perturbation of lipid membrane and interfacial tension may induce activity loss of membrane proteins [39], these SFLS results imply that the presence of hyper-osmotic stress can be more detrimental to the functional activity of influenza vaccine than hypo- or iso-osmotic stress at pH 2.0 and 37°C.

#### 2. Conformational change of antigenic proteins

**Figure 6:**
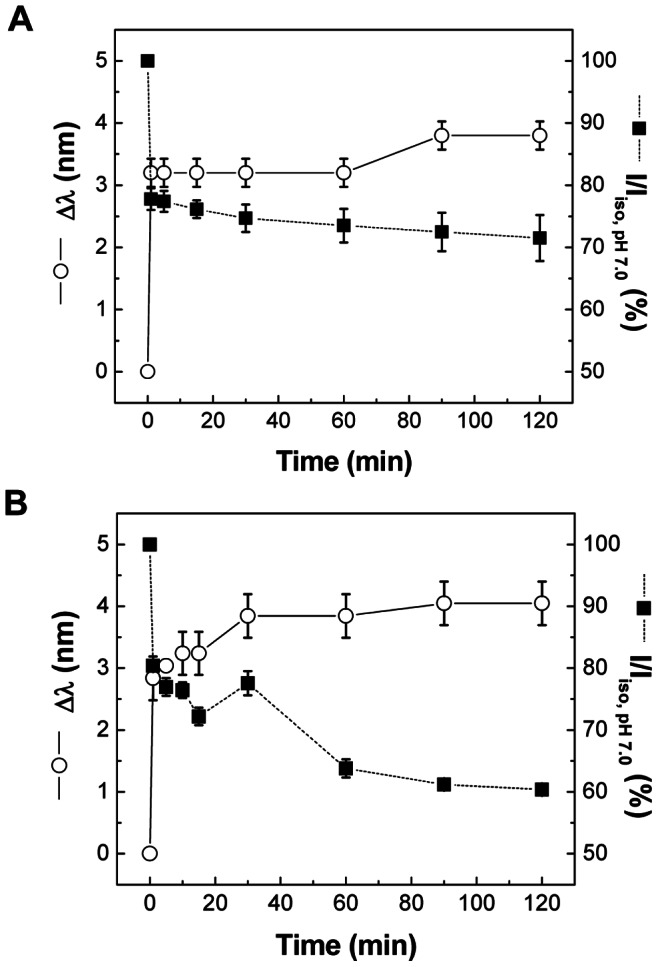
Intrinsic fluorescence spectra of influenza vaccine. Vaccine was incubated in (A) iso-osmotic medium and (B) hyper-osmotic medium (Δ = 300 mosM) at pH 7.0/2.0 and 37°C. Fluorescence spectra were measured after 1, 5, 15, 30, 60, 90, and 120 min of incubation, and the maximum emission position and intensity relative to control (pH 7.0) were plotted as a function of time. No significant fluorescence difference was observed between 4°C and 37°C. (Mean ± SD; *n* = 3.).

To evaluate conformational stability of antigenic proteins, intrinsic fluorescence was measured in the presence of iso- and hyper-osmotic stress at pH 2.0 and 37°C. The shifts in their maximum emission wavelength (Δλ = λ – λ_iso, pH 7.0_) and the relative intensity of emission maxima (I/I_iso, pH 7.0_) of influenza vaccine were measured in iso-osmotic medium at pH 2.0, in comparison with those in iso-osmotic solution at pH 7.0 at various time points (see Figures S3A and 6A for SFLS spectrum and fluorescence spectra analysis, respectively). As shown in Figure 6A, a considerable decrease in intensity (77.8±1.7%) and a red shift (Δλ = 3.2±0.2 nm) were observed after 1 min of exposure to iso-osmotic solution at pH 2.0. Longer incubation yielded further decrease of the relative intensity to 71.5±3.7% and increase of the peak shift (Δλ = 3.8±0.2 nm) after 2 h incubation. A major intensity decrease as well as red shift of the fluorescence emission spectrum within ∼ 1 min can be accounted for by an acid-induced conformational change in tertiary structure of antigenic proteins followed by additional small changes due to membrane destabilization. This observation is consistent with low pH-induced conformational changes of antigenic proteins such as trimeric HA proteins. That is, at low pH, a globular head domain (H1) containing the receptor binding sites is detrimerized and a stem domain (H2) undergoes sequential conformational changes by extruding fusion peptides [40]. Importantly, pH-induced conformational change of HA has been reported to accompany alteration of antigenic sites, thereby affecting immunogenicity [41]–[43].

With a hyper-osmotic gradient of 300 mosM, a large red shift of the intrinsic fluorescence after 1 min was followed by a progressive increase over the course of incubation time (see Figures S3C and 6B for SFLS spectrum and intrinsic fluorescence analysis, respectively). Exposure to hyper-osmotic stress (Figure 6B) significantly lowered the relative intensity of emission maxima compared to that of iso-osmotic condition as shown in Figure 6A (two-way ANOVA, *P*<0.005). As a result, 60.4±0.7% of I/I_iso, pH 7.0_ and 4.0±0.4 nm of Δλ were measured at 2 h. The red shift with intensity decrease at 1 min can be explained by pH-induced conformational change as in Figure 6A. However, considering the substantial amount of irregular scattering under hyper-osmotic stress (see Figure S3), it is reasonable to assume that the significantly enhanced peak shift and intensity decrease after 1 min was induced mainly by membrane perturbation and integrity loss. This observation is consistent with the previous prediction about functional activity decrease of influenza vaccine by osmotic stress-induced morphological change [33]. Therefore, intrinsic fluorescence data support our hypothesis that hyper-osmotic stress-induced morphological change of influenza vaccine made major contribution to the time-dependent activity loss of oral vaccine in gastric environment, subsequent to the rapid activity loss due to acid-induced protein denaturation at the initial stage within 1 min.

#### 3. Morphological change of vaccine

**Figure 7:**
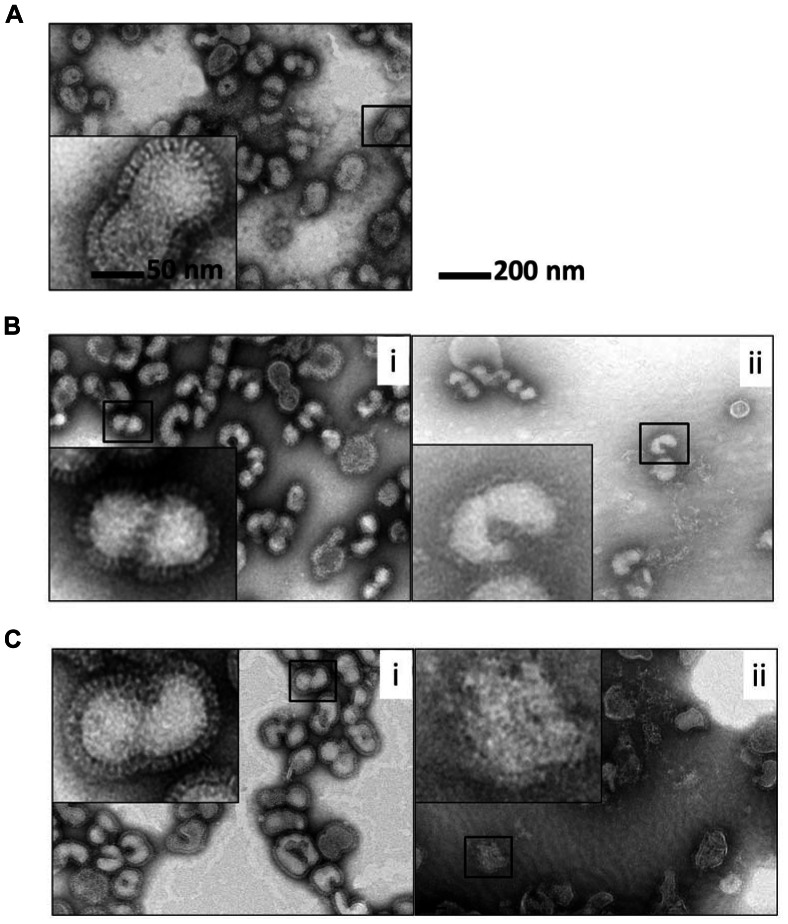
Negative-stain TEM micrographs of influenza vaccine. Vaccine was incubated in (A) iso-osmotic medium at pH 7.0 and 4°C, (B) iso-osmotic medium at (i) pH 7.0/(ii) pH 2.0 and 37°C, and (C) hyper-osmotic medium (Δ = 300 mosM) at (i) pH 7.0/(ii) pH 2.0 and 37°C for 2 h. Insets show magnified influenza vaccine images. After 2 h incubation, influenza vaccine was resuspended in PBS at pH 7.0 after centrifugation. The samples were stained with 2% phosphotungstic acid (pH 7.0) and allowed to dry under ambient conditions before imaging.

TEM analysis was performed to test vaccine destabilization mechanisms predicted by SFLS analysis and by intrinsic fluorescence. Influenza vaccine was exposed to Δ = 0 and 300 mosM at pH 7.0/2.0 and 37°C for 2 h, and their morphology was compared with that of the control (i.e., vaccine in iso-osmotic medium at 4°C). As shown in Figure 7, vaccine exposed to Δ = 0 and 300 mosM at pH 7.0 exhibited a similar morphology compared to the control (Figure 7A: control, Figure 7B(i): iso-osmotic, and Figure 7C(i): hyper-osmotic). Vaccine maintained its spherical shape and spike-like glycoproteins on the surface of the vaccine envelope. However, TEM micrograph in Figure 7B(ii) revealed that influenza vaccine in iso-osmotic acidic medium (pH 2.0) appeared to preserve spherical shape, however they did not display well-defined protrusion morphology, implying the loss of protein structure. This correlates well with our intrinsic fluorescence measurements in Figure 6. Moreover, upon exposure to hyper-osmotic acidic medium (Δ = 300 mosM, pH 2.0), the spherical shape of vaccine particles was partially lost and some of them had ruptured (Figure 7C(ii)), which is consistent with our SFLS analysis in Figure 5. Considering that vaccine suspension was neutralized after 2 h incubation and resuspended in iso-osmotic solution, the observed destructive effects of pH and osmotic stress on the morphology of virus are believed to be irreversible. Thus, TEM data support our hypothesis that vaccine destabilization in gastric environments is mainly associated with the hyper-osmotic stress-induced morphological change as well as the low pH-induced denaturation.

### Effect of Osmotic Stress on the Functional Activity of Influenza Vaccine at pH 2.0 and 37°C

**Figure 8:**
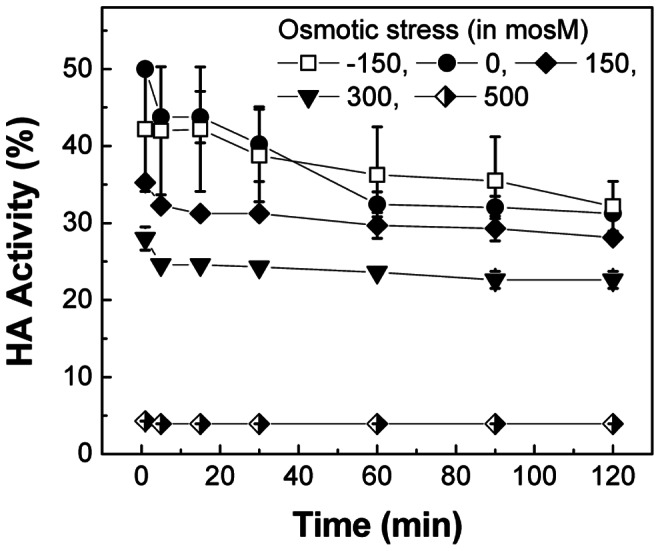
Effects of pH on functional activity of influenza vaccine. Hemagglutinating activity of vaccine in osmolyte solution (Δ = −150, 0, 150, 300, and 500 mosM) was measured after 1, 5, 15, 30, 60, 90, and 120 min of incubation at pH 2.0 and 37°C, and the remaining activity relative to control (pH 7.0 and 4°C) is plotted as a function of time. No HA activity decrease was observed by increasing temperature to 37°C (pH 7.0) during measurements. (Mean ± SD; *n* = 8–16.).

The effect of osmotic stress on the functional HA activity of influenza vaccine was examined by measuring hemagglutinating titer in the presence of various osmotic stresses (Δ = −150, 0, 150, 300, and 500 mosM) at pH 2.0 and 37°C. In contrast to ∼ 10% of HA activity loss upon exposure to Δ = 500 mosM at pH 7.0 and 37°C, no significant level of functional HA activity loss was observed from vaccine after 2 h incubation at Δ = −150, 0, 150, and 300 mosM (data not shown). However, as shown in Figure 8, upon exposure to iso-osmotic acidic solution, vaccine lost about 50% of HA activity after 1 min, and 68% of HA activity after 2 h. From the analyses of SFLS and intrinsic fluorescence, the initial activity loss can be explained by the low pH-induced conformational change of antigenic proteins (as shown in Figure 6A), and the further decrease in HA activity during incubation by the combined effects of dilution and low pH-induced aggregation (as discussed in Figure 5B). Interestingly, no significant difference in the time-dependent HA activity change was observed between iso- and hypo-osmotic stress (two-way ANOVA, *P* = 0.504). This indicates that morphological change due to swelling does not exert deteriorative effect on the functional activity of vaccine. In contrast to iso- and hypo-osmotic stress, exposure to hyper-osmotic condition led to significant decrease in the magnitude of functional HA activity with increasing magnitude of osmotic stress (two-way ANOVA, *P*<0.001). As a result, 35, 27, and 4% of the remaining HA activity was measured at Δ = 150, 300, and 500 mosM, respectively, after 1 min of incubation. The decrease in the functional HA activity with increasing hyper-osmotic stress and almost complete loss of functional HA activity at Δ = 500 mosM can be associated with the membrane integrity loss and disruption, as demonstrated by TEM analysis; the increase in deformation with increasing hyper-osmotic stress can be seen from the comparison of TEM micrographs (Figures 7B(ii), S4A, 7C(ii), and S4B for Δ = 0, 150, 300, and 500 mosM, respectively). Considering the fact that H1 subunit serves as a receptor by binding to sialic acid (N-acetylneuraminic acid) of host cells, the decrease of HA activity indicates that low pH treatment and osmotic pressure induced conformational changes in H1 and/or the receptor binding sites, supporting our hypothesis that osmotic stress-induced structural destabilization as well as pH-induced conformational change of antigenic proteins of influenza vaccine contributed to the decrease in HA activity under acidic environments.

## Discussion

While gastric acid-induced vaccine activity loss has long been an important question, there is still very little systematic understanding of vaccine destabilization process in a physiological gastric environment. Instead, a great deal of research has been focused to develop a new vaccine delivery system to increase efficacy of oral vaccine. Quantitative assessment of oral influenza vaccine stability in gastric environment has been hampered by a lack of tools to monitor the combined and/or separate influences of temperature, pH, and osmotic stress and on the functional activity of influenza vaccine as a function of time. For this purpose, we employed SFLS analysis to monitor time-dependent morphological change of influenza vaccine and thus to study the role of each environmental factor involved in gastric digestion process.

### PC-liposome as a Model for SFLS Analysis of Influenza Vaccine

Embryonated hen’s eggs have been used as the substrate for the production of currently licensed influenza vaccine [6]. Since influenza virus replicates in the cells consisting of chorioallantoic membrane, A/PR/8/34 influenza virus incorporates lipid components present in the host cells of the egg including phosphatidylcholine, sphingomyelin, phosphatidylethanolamine, phosphatidylserine, etc [25], [26]. However, it is not practical to identify precise lipid composition of the strain-specific, host-cell dependent influenza virus. Subsequently the difference in phospholipid composition between vaccine and liposome may produce non-identical osmotic swell-shrink behavior because lipid composition has been known to influence the rigidity and the water permeability of the membrane [44], [45]. An important feature of influenza vaccine is that its wall can be seen as a double-shelled composite structure consisted of the M1 protein shell and lipid bilayers that have additional intermolecular interactions causing different mechanical properties from single-walled liposomes. Another factor that should be noted is that we used a linear correlation between osmotic stress and liposome volume assuming that the liposome behaves similarly to a perfect osmometer [46]. However, while this approximation may work well for relatively high osmotic pressure, it may not be suitable for low osmotic stress, possibly because of opposing activities of membrane tensile strength or compressive strength to osmotic swell-shrink behavior [47]. Although further optimization steps are needed to develop reference liposomes, our approach, assuming the same mechanical properties between influenza virus and liposomes, using SFLS as a guide has been particularly useful in predicting time-dependent morphological change of influenza vaccine in response to gastric environmental factors.

### Temperature-pH-osmotic Stress

To investigate the effects of physiological temperature (37°C), the hyper-osmotic shrinking behavior of influenza vaccine was characterized. Osmolyte solution was controlled to generate hyper-osmotic stress of 300 mosM and SFLS measurements were performed at five different temperatures. As shown in Figures 3B and 3D, influenza vaccine exhibited a step-wise size change at both pH 7.0 and 2.0. At pH 7.0, regardless of temperature, a small but rapid shrinkage (V_rel_ ∼ 90%) in the first shrinkage stage was accompanied by a large but slow shrinkage (V_rel_ ∼ 45%) in the secondary shrinkage stage. The first was considered to cause an increase in the packing density of the viral membrane and the latter was due to the resistance of the M1 shell to further shrinkage [33]. Since the shrinkage process is driven by osmotic stress, saturation of V_rel_ would depend on the size and direction of the applied osmotic stress, which explains why similar levels of V_rel_ were measured at different temperatures. On the other hand, the temperature increase resulted in the faster first shrinkage and the earlier initiation of the secondary shrinkage, as evidenced by increasing *k* and decreasing t_2nd_ with the increase of temperature (Figures 3E and 3F). Both *k* increase and t_2nd_ decrease can be explained by higher water efflux across the membrane due to increased thermal energy and/or by reduced intermolecular associations.

At pH 2.0, influenza vaccine experienced a net size increase during the initial stage of volume change, probably due to low pH-induced aggregation. This was supported by the absence of evidence for virus-virus fusion from SFLS spectra. Vaccine clustering occurred more dominantly with increasing temperatures, so that clearly observable hyper-osmotic vaccine shrinkage was not detected from SFLS spectra at pH 2.0 and ≥25°C (Figure 3D). 8-s scan SFLS spectra (Figure 3C(i)) at 25°C (pH 2.0) showed the evidence for the competing influences of hyper-osmotic shrinkage and aggregation. Therefore, it is predicted that dominant contributions to the total scattering ≥25°C came from the clustering of the shrunk vaccine, whereas hyper-osmotic stress-induced step-wise shrinkage is due to insignificant aggregation at pH 2.0 and 4°C. That is, aggregation reaction is considered as a general feature of vaccine at low pH and was faster at high temperatures. This is further supported by the decrease of light scattering intensity for all osmotic stresses, including hyper-osmotic stress, at pH 2.0 and 37°C (Figure 4).

SFLS analysis was performed for 2 h to better understand osmotic stress-induced destabilization of influenza vaccine over a similar time course to gastric digestion. As implied by SFLS analysis in Figures S2 and 5A, influenza vaccine at pH 7.0 exhibited its typical step-wise morphological change, similarly to short-term scans. After 2 h of incubation, in response to Δ = −150, 150, and 300 mosM, the volume was changed to 122, 58, and 32% relative to that of vaccine in iso-osmotic solution; in other words, 107, 83, and 68% relative to the radius of vaccine in iso-osmotic solution. In the case of iso-osmotic condition, the size of influenza vaccine gradually increased to V_rel_ ∼ 112% (104% in radius) in 2 h.

At pH 2.0, the previously mentioned clustering tendency was more strongly displayed for all osmotic stresses regardless of their direction (Δ = −150, 0, 150, and 300 mosM) during the first phase of size change beginning at 0 s. Due to the contribution of low pH-induced aggregation, incubation of vaccine in iso-osmotic condition for 2 h yielded an additional 24% increase in the relative volume of the vaccine aggregate (136% in V_rel_ and 111% in radius). Since aggregated vaccine exposes less binding sites in a non-uniform fashion, influenza vaccine is expected to lose hemagglutinating activity over the progress of aggregation at acidic pH.

As shown in Figures 4B and 5B, influenza vaccine exhibited a typical hypo-osmotic behavior at Δ = −150 mosM; i.e., a step-wise swelling of vaccine to 146% in V_rel_ (113% in radius). On the other hand, in the presence of hyper-osmotic gradients (150 and 300 mosM), vaccine exposed to hyper-osmotic stress was easily destabilized indicating morphological change, membrane integrity loss, and/or defect formation by SFLS analysis (Figures S3 and 5B). From TEM analysis, we found that the vaccine envelope is destabilized in an irreversible fashion with the increase of hyper-osmotic stress at pH 2.0 (Figures 7 and S4). Since antigenic proteins are embedded in lipid membrane, a significant perturbation or disruption of the membrane can impair their stability. Indeed, our intrinsic fluorescence data demonstrated that antigenic proteins of influenza vaccine experienced more conformational changes in hyper-osmotic gradient (Figure 6B) than in iso-osmotic gradient (Figure 6A). The observed decreases in HA activity with increasing hyper-osmotic gradient support theses analyses. The results further suggest that the deteriorative temperature and pH effects were significantly amplified by hyper-osmotic shock.

The conformational stability of HA proteins plays a critical role in the changes of infectivity and immunogenicity at low pH. That is, low pH-induced structural change in trimer interface affects the receptor binding activity of HA as well as the interaction with immune cells such as B lymphocytes and antigen-presenting cells, thus affecting broad immune response [41], [48], [49]. Although there is a difference in the binding sites for sialic acid and antibodies, the similar effect of a single amino acid substitution on both receptor-binding specificity and monoclonal antibody-binding property has been ascribed to their proximity [41], [50]. This accounts for previous reports showing positive correlation between HA activity and immunogenicity [13], [51]–[53]. Therefore, in this work hemagglutination activity was used as a simple indicator of vaccine activity (i.e., immunogenicity). Despite of the necessity of *in vivo* studies, observations of HA activity change, intrinsic fluorescence, SFLS, and TEM analyses indicate that low pH-induced denaturation of antigenic proteins and aggregation play a critical role in the initial rapid vaccine activity loss, and morphological change of vaccine causes additional activity decrease during further incubation under hyper-osmotic conditions.

### Gastric Digestion

In this study, stability of influenza vaccine in gastric environments was investigated under simplified conditions using SFLS method. Sucrose was used as the osmolyte to apply osmotic stress to influenza vaccine, because it is easy to generate an osmotic gradient across viral membrane due to the impermeability of the virus envelope to sugar. The use of sucrose proved to be a highly effective method to predict how the influenza vaccine behaves under various osmotic stress environments. However, considering the presence of ions, digestive enzymes, and nutrients and gastric motility during digestion [54], [55], the combined and individual effects of those factors on the osmotic stability of influenza vaccine deserves further investigation.

Another important factor not dealt with in sufficient detail in this work, is dilution-induced vaccine destabilization. As shown in Figure S5, a highly diluted vaccine was proven to be more susceptible to osmotic stress-induced HA activity loss (two-way ANOVA, *P*<0.001), which is consistent with a previous report [56]. Even at iso-osmotic condition, highly diluted vaccine exhibited a significantly lower HA activity than a relatively less diluted one (Student’s *t*-test, *P*<0.001). It was also predicted that the size increase of influenza vaccine at iso-osmotic condition and pH 7.0 would be associated with the dilution-induced destabilization (Figure 5A). Thus, while further research is needed to find the mechanisms, it is clear that dilution plays an important role in determining stability of vaccine during gastric digestion process.

Osmotic pressure during digestion is influenced by various factors such as stomach content and gastric secretions due to ingestion and stimulatory/inhibitory digestive functions [57]. One would expect that there is not a consistent measure of the gastric osmolarity due to variability in meal-to-meal and person-to-person differences. In addition, it was reported that gastric osmolarity affects gastric emptying time; as a result, the rate of gastric emptying was slowed in the presence of hyper-osmotic pressure [58]. Thus, influenza vaccine can remain in the stomach for longer periods at hyper-osmotic condition than hypo- or iso-osmotic conditions. As predicted from our analysis in this work, a maximum level of stability can be maintained at both hypo- and iso-osmotic gastric conditions, but hyper-osmotic conditions result in a minimum level of activity. It is expected that a longer gastric residence time at hyper-osmotic condition would make influenza vaccine much more unstable in the strong acidic environment of stomach. Therefore, our study suggests that in order to maintain a high level of vaccine efficacy, it is critical to take an oral influenza vaccine on an empty stomach and to develop a formulation that can protect vaccine against hyper-osmotic pressure as well as low pH in the gastric environment.

### Conclusions

In this work, we investigated destabilization mechanisms of whole inactivated influenza virus vaccine in gastric digestion environment using SFLS, focusing on the demonstration of how temperature, pH, and osmotic pressure affect the stability of vaccine during digestion process. Time-dependent morphological change of vaccine was predicted by SFLS analysis under diverse testing conditions and its validity and applicability was confirmed with TEM, intrinsic fluorescence, and hemagglutinating activity. At iso-osmotic, acidic, and physiological temperature conditions, about 50% of vaccine HA activity loss was observed within 1 min. This was associated with low pH-induced conformational change of antigenic proteins. A further decrease to 32% of the remaining HA activity with up to 2 h incubation was hypothesized to come from the combined effects of dilution and pH. Vaccine exposed to hypo-osmotic stress displayed a similar time-dependent activity decrease as in iso-osmotic conditions, indicating that swelling does not induce any significant change in antigenic activity. We found that the presence of hyper-osmotic stress in acidic medium had a significant destabilizing effect on vaccine stability. The extent of irreversible membrane deformation increased with the increase of osmotic stress, which caused additional denaturation of the antigenic proteins. Further, low pH-induced aggregation was more dominantly observed in acidic hyper-osmotic solution than in acidic iso-osmotic solution. A marked destabilization of vaccine was observed at hyper-osmotic stress ≥300 mosM, and as a result, no significant level of vaccine activity was observed at 500 mosM at pH 2.0 and 37°C. Although only three representative gastric environmental factors (pH, temperature, and osmotic stress) were discussed in this study, SFLS analysis has provided new insights into mechanisms of oral vaccine damage during digestion. This research is expected to contribute to the development of oral vaccine formulations for whole inactivated influenza virus as well as other envelope vaccines.

## Supporting Information

Figure S1 **Osmotic stress dependence of the relative light scattering intensity (I_rel_) of PC-liposomes.** (Mean ± SD; *n* = 54.) The plot was fitted by a double-exponential curve to find the correlation: I_rel_ = 1+ c·e^x/Δ^+d·e^y/Δ^.
(TIF)

Figure S2 **Long-term course of SFLS curves of influenza vaccine exposed to osmotic stress of −150, 0, 150, and 300 mosM at pH 7.0 and 37°C.** Spectra are representative of *n* = 9 replicate samples examined at each condition.
(TIF)

Figure S3 (A) Long-term course of SFLS curves of influenza vaccine exposed to osmotic stress of −150, 0, 150, and 300 mosM at pH 2.0 and 37°C. Magnified SFLS spectra (I_rel_) and V_rel_ corresponding to (B) Δ = 150 mosM and (C) Δ = 300 mosM. Spectra are representative of *n* = 9 replicate samples examined at each condition.
(TIF)

Figure S4 **Negative-stain TEM images of influenza vaccine.** Vaccine exposed to hyper-osmotic stress of (A) 150 mosM and (B) 500 mosM at pH 2.0 and 37°C for 2 h.
(TIF)

Figure S5 **Effects of dilution on the functional HA activity of influenza vaccine.** The vaccine stock was 10- and 50-fold diluted to make final concentration of 0.5 mg/ml in three different osmotic media, i.e. (A) iso-osmotic, (B) 300 mosM, and (C) 500 mosM, at pH 7.0 and 37°C. HA activity was measured after 2 h incubation at 37°C and plotted relative to that of control sample (vaccine in iso-osmotic medium at 4°C) before dilution. (Mean ± SD; *n* = 8).
(TIF)

## References

[1] Thompson WW, Shay DK, Weintraub E, Brammer L, Cox N, et al (2003) Mortality associated with influenza and respiratory syncytial virus in the United States. JAMA 289: 179–186. 12517228 10.1001/jama.289.2.179

[2] Fauci AS (2006) Emerging and re-emerging infectious diseases: Influenza as a prototype of the host-pathogen balancing act. Cell 124: 665–670. 16497575 10.1016/j.cell.2006.02.010 PMC7126645

[3] Molinari NA, Ortega-Sanchez IR, Messonnier ML, Thompson WW, Wortley PM, et al (2007) The annual impact of seasonal influenza in the US: measuring disease burden and costs. Vaccine 25: 5086–5096. 17544181 10.1016/j.vaccine.2007.03.046

[4] Harper SA, Fukuda K, Uyeki TM, Cox NJ, Bridges CB (2004) Prevention and control of influenza: recommendations of the advisory committee on immunization practices (ACIP). MMWR Recomm Rep 53: 1–40. 15163927

[5] Fiore AE, Uyeki TM, Broder K, Finelli L, Euler GL, et al (2010) Prevention and control of influenza with vaccines: recommendations of the advisory committee on immunization practices (ACIP). MMWR Recomm Rep 59: 1–62. 20689501

[6] Keitel WA, Couch RB (2002) Inactivated influenza vaccines. In: Potter CW, editor. Perspectives in Medical Virology: Elsevier. 145–177.

[7] Giudice EL, Campbell JD (2006) Needle-free vaccine delivery. Adv Drug Deliv Rev 58: 68–89. 16564111 10.1016/j.addr.2005.12.003

[8] Neutra MR, Kozlowski PA (2006) Mucosal vaccines: the promise and the challenge. Nat Rev Immunol 6: 148–158. 16491139 10.1038/nri1777

[9] Palese P (2006) Making better influenza virus vaccines? Emerg Infect Dis 12: 61–65. 16494719 10.3201/eid1201.051043 PMC3291403

[10] Mutsch M, Zhou WG, Rhodes P, Bopp M, Chen RT, et al (2004) Use of the inactivated intranasal influenza vaccine and the risk of Bell's palsy in Switzerland. N Engl J Med 350: 896–903. 14985487 10.1056/NEJMoa030595

[11] Farag-Mahmod FI, Wyde PR, Rosborough JP, Six HR (1988) Immunogenicity and efficacy of orally administered inactivated influenza virus vaccine in mice. Vaccine 6: 262–268. 3420975 10.1016/0264-410x(88)90222-8

[12] Lazzell V, Waldman RH, Rose C, Khakoo R, Jacknowitz A, et al (1984) Immunization against influenza in humans using an oral enteric-coated killed virus-vaccine. J Biol Stand 12: 315–321. 6480615 10.1016/s0092-1157(84)80012-8

[13] Quan FS, Li ZN, Kim MC, Yang D, Compans RW, et al (2011) Immunogenicity of low-pH treated whole viral influenza vaccine. Virology 417: 196–202. 21722934 10.1016/j.virol.2011.05.014 PMC3152636

[14] Azizi A, Kumar A, Diaz-Mitoma F, Mestecky J (2010) Enhancing oral vaccine potency by targeting intestinal M cells. PLoS Pathog 6: e1001147. 21085599 10.1371/journal.ppat.1001147 PMC2978714

[15] Shastri PN, Kim MC, Quan FS, D'Souza MJ, Kang SM (2012) Immunogenicity and protection of oral influenza vaccines formulated into microparticles. J Pharm Sci 101: 3623–3635. 22711602 10.1002/jps.23220 PMC5558794

[16] Rose EF (1979) Factors influencing gastric emptying. J Forensic Sci 24: 200–206. 512606

[17] Hellmig S, Von Schoning F, Gadow C, Katsoulis S, Hedderich J, et al (2006) Gastric emptying time of fluids and solids in healthy subjects determined by 13C breath tests: influence of age, sex and body mass index. J Gastroenterol Hepatol 21: 1832–1838. 17074022 10.1111/j.1440-1746.2006.04449.x

[18] Ong BY, Palahniuk RJ, Cumming M (1978) Gastric volume and pH in out-patients. Can Anaesth Soc J 25: 36–39. 23891 10.1007/BF03006781

[19] Schwarz H, Koch AL (1995) Phase and electron microscopic observations of osmotically induced wrinkling and the role of endocytotic vesicles in the plasmolysis of the Gram-negative cell wall. Microbiology 141: 3161–3170. 8574409 10.1099/13500872-141-12-3161

[20] Scheie PO (1969) Plasmolysis of Escherichia Coli B/R with Sucrose. J Bacteriol 98: 335–340. 4891252 10.1128/jb.98.2.335-340.1969 PMC284818

[21] Waite MR, Pfefferkorn ER (1968) Effect of altered osmotic pressure on the growth of Sindbis virus. J Virol 2: 759–760. 5723530 10.1128/jvi.2.7.759-760.1968 PMC375685

[22] Colwell W, Simmons D, Harris J, Fulp T, Carrozza J, et al.. (1975) Influence of some physical factors on survival of Marek's disease vaccine virus. Avian Dis: 781–790. 987

[23] Skehel JJ, Bayley PM, Brown EB, Martin SR, Waterfield MD, et al (1982) Changes in the conformation of influenza-virus hemagglutinin at the pH optimum of virus-mediated membrane-fusion. Proc Natl Acad Sci U S A 79: 968–972. 6951181 10.1073/pnas.79.4.968 PMC345880

[24] Bullough PA, Hughson FM, Skehel JJ, Wiley DC (1994) Structure of influenza haemagglutinin at the pH of membrane fusion. Nature 371: 37–43. 8072525 10.1038/371037a0

[25] Kates M, Allison A, Tyrrell D, James A (1961) Lipids of influenza virus and their relation to those of the host cell. Biochim Biophys Acta 52: 455–466.

[26] Blough HA (1971) Fatty acid composition of individual phospholipids of influenza virus. J Gen Virol 12: 317–320. 4330348 10.1099/0022-1317-12-3-317

[27] Quan FS, Compans RW, Nguyen HH, Kang SM (2008) Induction of heterosubtypic immunity to influenza virus by intranasal immunization. J Virol 82: 1350–1359. 18032492 10.1128/JVI.01615-07 PMC2224423

[28] Woodle MC, Papahadjopoulos D (1989) [9] Liposome preparation and size characterization. In: Sidney Fleischer BF, editor. Method Enzymol: Academic Press. 193–217. 10.1016/s0076-6879(89)71012-0 2593841

[29] Lammert AM, Schmidt SJ, Day GA (1998) Water activity and solubility of trehalose. Food Chem 61: 139–144.

[30] Malmberg CG, Maryott AA (1950) Dielectric constants of aqueous solutions of dextrose and sucrose. J Res Nat Bur Stand 45: 299–303.

[31] Choi HJ, Ebersbacher CF, Quan FS, Montemagno CD (2013) pH stability and comparative evaluation of ranaspumin-2 foam for application in biochemical reactors. Nanotechnology 24: 055603. 23324183 10.1088/0957-4484/24/5/055603

[32] Sun ST, Milon A, Tanaka T, Ourisson G, Nakatani Y (1986) Osmotic swelling of unilamellar vesicles by the stopped-flow light-scattering method -elastic properties of vesicles. Biochim Biophys Acta 860: 525–530.

[33] Choi HJ, Bondy BJ, Yoo DG, Compans RW, Kang SM, et al (2013) Stability of whole inactivated influenza virus vaccine during coating onto metal microneedles. J Control Release 166: 159–171. 23246470 10.1016/j.jconrel.2012.12.002 PMC3578180

[34] Kim CH, Macosko JC, Shin YK (1998) The mechanism for low-pH-induced clustering of phospholipid vesicles carrying the HA2 ectodomain of influenza hemagglutinin. Biochemistry 37: 137–144. 9425033 10.1021/bi971982w

[35] Brown JD, Goekjian G, Poulson R, Valeika S, Stallknecht DE (2009) Avian influenza virus in water: Infectivity is dependent on pH, salinity and temperature. Vet Microbiol 136: 20–26. 19081209 10.1016/j.vetmic.2008.10.027

[36] Rich GT, Sha'afi I, Romualdez A, Solomon AK (1968) Effect of osmolality on the hydraulic permeability coefficient of red cells. J Gen Physiol 52: 941–954. 5722087 10.1085/jgp.52.6.941 PMC2225854

[37] Fatouros DG, Antimisiaris SG (2002) Effect of amphiphilic drugs on the stability and zeta-potential of their liposome formulations: A study with prednisolone, diazepam, and griseofulvin. J Colloid Interf Sci 251: 271–277. 10.1006/jcis.2002.8432 16290730

[38] Fatouros DG, Antimisiaris SG (2001) Physicochemical properties of liposomes incorporating hydrochlorothiazide and chlorothiazide. J Drug Target 9: 61–74. 11378524 10.3109/10611860108995633

[39] Elliott JR, Needham D, Dilger JP, Haydon DA (1983) The effects of bilayer thickness and tension on gramicidin single-channel lifetime. Biochim Biophys Acta 735: 95–103. 6194820 10.1016/0005-2736(83)90264-x

[40] Skehel J, Bizebard T, Bullough P, Hughson F, Knossow M, et al. Membrane fusion by influenza hemagglutinin; 1995. Cold Spring Harbor Laboratory Press. 573–580. 10.1101/sqb.1995.060.01.061 8824430

[41] Wiley DC, Skehel JJ (1987) The structure and function of the hemagglutinin membrane glycoprotein of influenza virus. Annu Rev Biochem 56: 365–394. 3304138 10.1146/annurev.bi.56.070187.002053

[42] Daniels R, Douglas A, Skehel J, Wiley D (1983) Analyses of the antigenicity of influenza haemagglutinin at the pH optimum for virus-mediated membrane fusion. J Gen Virol 64: 1657–1662. 6192202 10.1099/0022-1317-64-8-1657

[43] Yewdell JW, Gerhard W, Bachi T (1983) Monoclonal anti-hemagglutinin antibodies detect irreversible antigenic alterations that coincide with the acid activation of influenza-virus A/PR/834-mediated hemolysis. J Virol 48: 239–248. 6193286 10.1128/jvi.48.1.239-248.1983 PMC255340

[44] Milon A, Lazrak T, Albrecht AM, Wolff G, Weill G, et al (1986) Osmotic swelling of unilamellar vesicles by the stopped-flow light scattering method. Influence of vesicle size, solute, temperature, cholesterol and three α, ω-dihydroxycarotenoids. Biochim Biophys Acta 859: 1–9.

[45] Bittman R, Leventhal AM, Karp S, Blau L, Tremblay PA, et al (1981) Osmotic behavior of liposomes of phosphatidylcholine and phosphatidylsulfocholine as a function of lipid-concentration. Chem Phys Lipids 28: 323–335.

[46] Ponder E (1944) The osmotic behavior of crenated red cells. J Gen Physiol 27: 273–285. 19873386 10.1085/jgp.27.4.273 PMC2238020

[47] Knowles CJ, Smith L (1971) Effect of osmotic pressure of the medium on the volume of intact cells of Azotobacter vinelandii and on the rate of respiration. Biochim Biophys Acta 234: 144–152. 4934673 10.1016/0005-2728(71)90139-3

[48] Staneková Z, Vareckova E (2010) Conserved epitopes of influenza A virus inducing protective immunity and their prospects for universal vaccine development. Virol J 7: 351. 21118546 10.1186/1743-422X-7-351 PMC3009981

[49] Cox RJ, Brokstad KA, Ogra P (2004) Influenza virus: immunity and vaccination strategies. Comparison of the immune response to inactivated and live, attenuated influenza vaccines. Scand J Immunol 59: 1–15. 14723616 10.1111/j.0300-9475.2004.01382.x

[50] Daniels RS, Douglas AR, Skehel JJ, Wiley DC, Naeve CW, et al (1984) Antigenic analyses of influenza virus haemagglutinins with different receptor-binding specificities. Virology 138: 174–177. 6208680 10.1016/0042-6822(84)90158-2

[51] Choi HJ, Yoo DG, Bondy BJ, Quan FS, Compans RW, et al (2012) Stability of influenza vaccine coated onto microneedles. Biomaterials 33: 3756–3769. 22361098 10.1016/j.biomaterials.2012.01.054 PMC3586192

[52] Quan FS, Kim YC, Vunnava A, Yoo DG, Song JM, et al (2010) Intradermal vaccination with influenza virus-like particles by using microneedles induces protection superior to that with intramuscular immunization. J Virol 84: 7760–7769. 20484519 10.1128/JVI.01849-09 PMC2897640

[53] Quan FS, Kim YC, Yoo DG, Compans RW, Prausnitz MR, et al (2009) Stabilization of influenza vaccine enhances protection by microneedle delivery in the mouse skin. PLoS One 4: e7152. 19779615 10.1371/journal.pone.0007152 PMC2745577

[54] Szarka LA, Camilleri M (2009) Methods for measurement of gastric motility. Am J Physiol Gastrointest Liver Physiol 296: G461–475. 19147807 10.1152/ajpgi.90467.2008

[55] Hunt JN (1959) Gastric emptying and secretion in man. Physiol Rev 39: 491–533. 13674903 10.1152/physrev.1959.39.3.491

[56] Miller GL (1944) Influence of pH and of certain other conditions on the stability of the infectivity and red cell agglutinating activity of influenza virus. J Exp Med 80: 507–520. 19871433 10.1084/jem.80.6.507 PMC2135484

[57] Hunt JN (1959) Gastric emptying and secretion in man. Physiol Rev 39: 491–533. 13674903 10.1152/physrev.1959.39.3.491

[58] McSwiney B, Spurrell W (1933) Influence of osmotic pressure upon the emptying time of the stomach. J Physiol 79: 437–442. 16994470 10.1113/jphysiol.1933.sp003057 PMC1394854

