# Supplementary figures and images for: A Mechanistic Study on the Destabilization of Whole Inactivated Influenza Virus Vaccine in Gastric Environment
Source: PLoS One. 2013 Jun 11;8(6):e66316. doi: 10.1371/journal.pone.0066316 (PMC3679046; doi:10.1371/journal.pone.0066316)

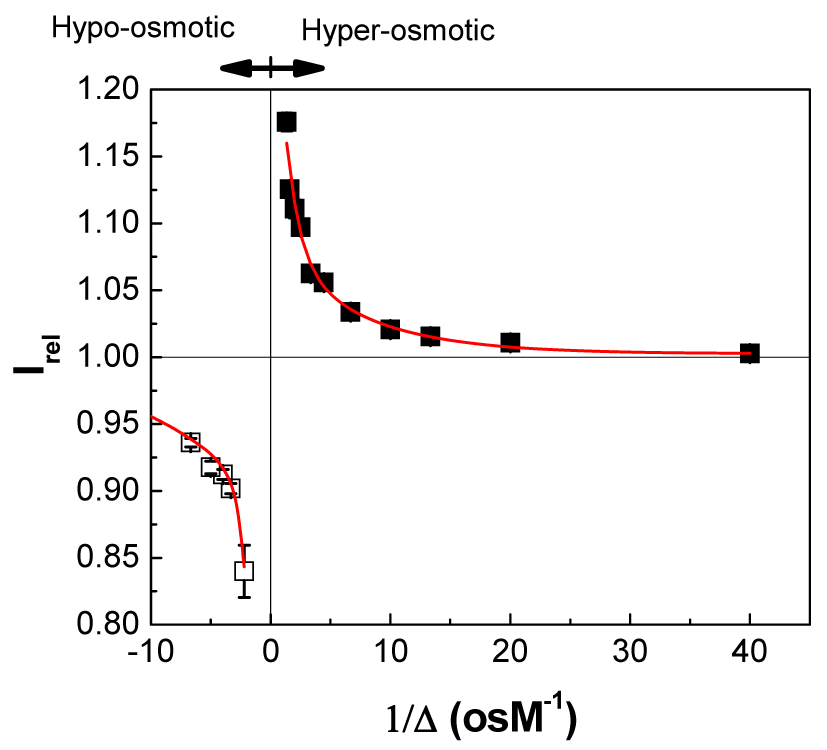

Supplement: Figure S1 — Osmotic stress dependence of the relative light scattering intensity (Irel) of PC-liposomes. (Mean ± SD; n = 54.) The plot was fitted by a double-exponential curve to find the correlation: Irel = 1+ c·ex/Δ+d·ey/Δ. (TIF) [file pone.0066316.s001.tif]

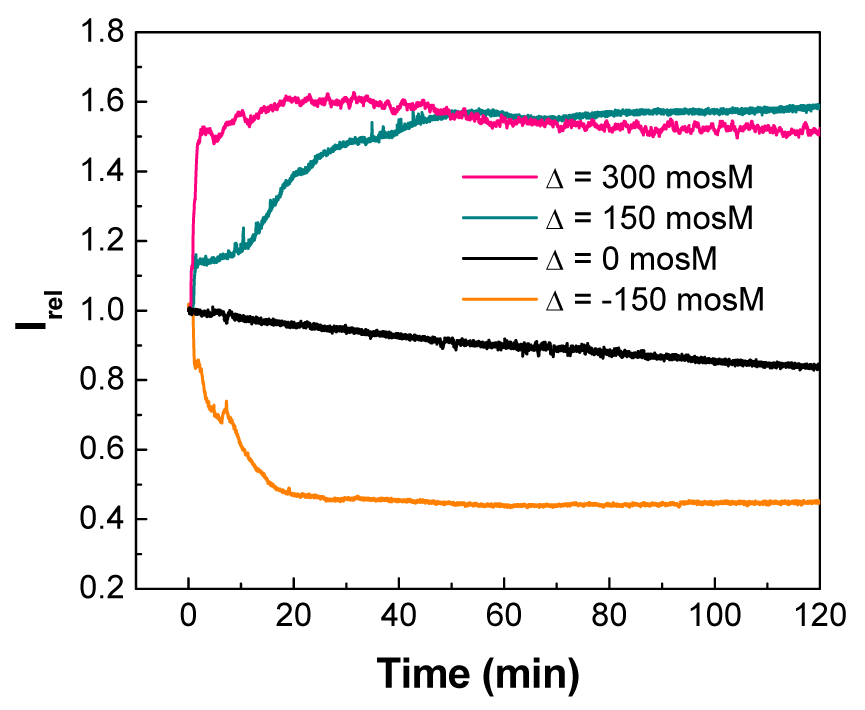

Supplement: Figure S2 — Long-term course of SFLS curves of influenza vaccine exposed to osmotic stress of −150, 0, 150, and 300 mosM at pH 7.0 and 37°C. Spectra are representative of n = 9 replicate samples examined at each condition. (TIF) [file pone.0066316.s002.tif]

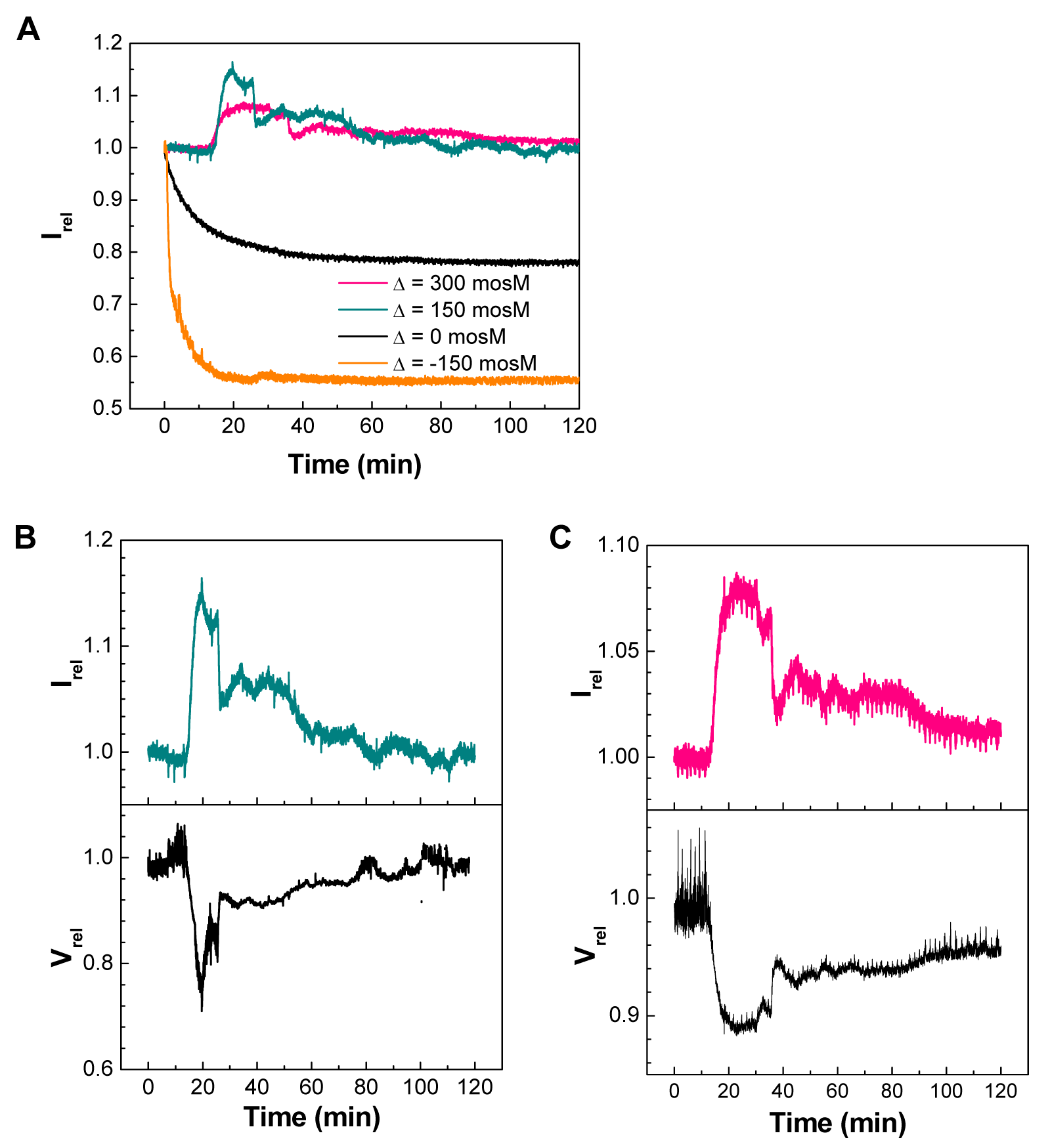

Supplement: Figure S3 — (A) Long-term course of SFLS curves of influenza vaccine exposed to osmotic stress of −150, 0, 150, and 300 mosM at pH 2.0 and 37°C. Magnified SFLS spectra (Irel) and Vrel corresponding to (B) Δ = 150 mosM and (C) Δ = 300 mosM. Spectra are representative of n = 9 replicate samples examined at each condition. (TIF) [file pone.0066316.s003.tif]

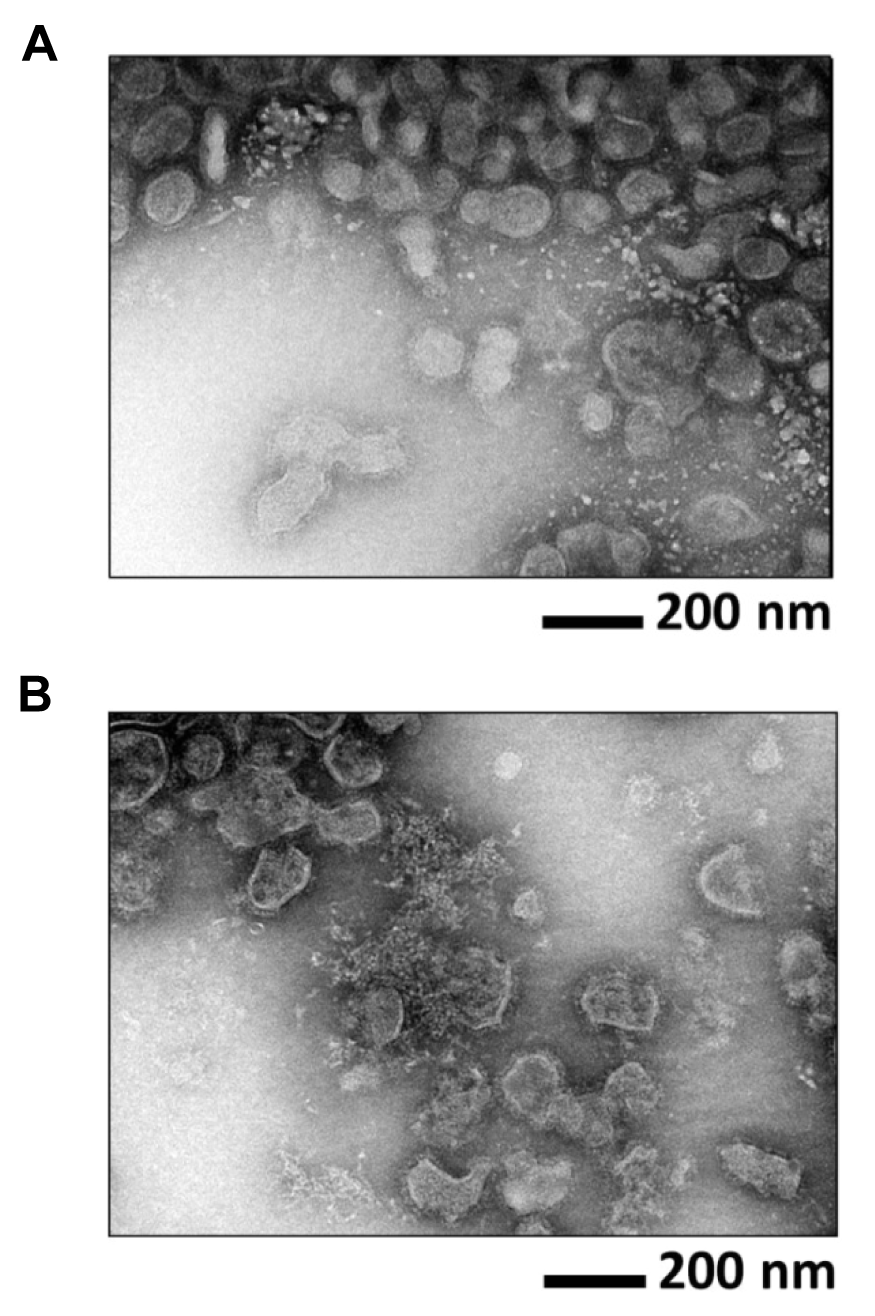

Supplement: Figure S4 — Negative-stain TEM images of influenza vaccine. Vaccine exposed to hyper-osmotic stress of (A) 150 mosM and (B) 500 mosM at pH 2.0 and 37°C for 2 h. (TIF) [file pone.0066316.s004.tif]

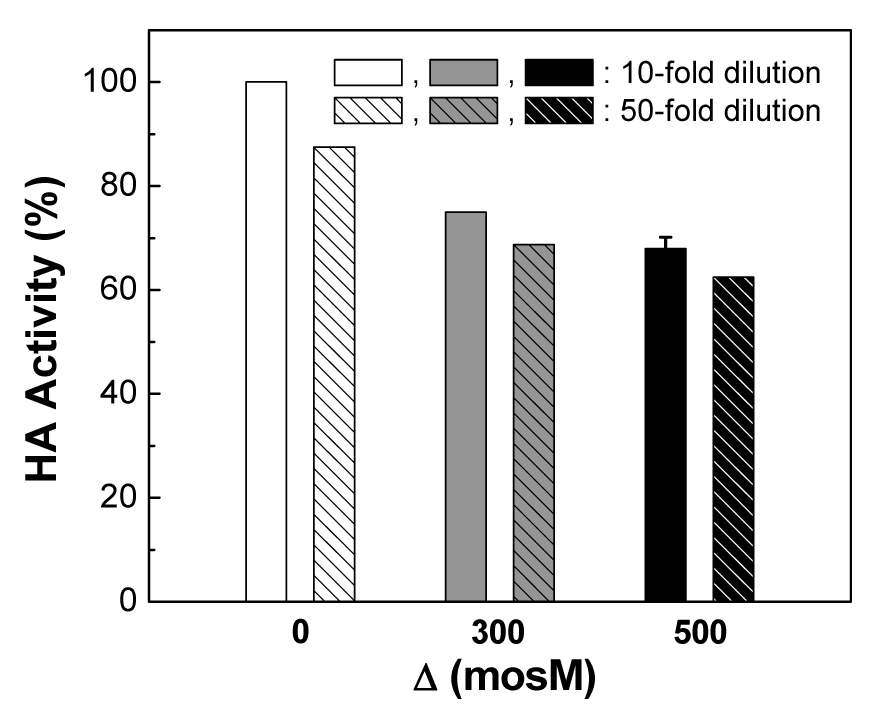

Supplement: Figure S5 — Effects of dilution on the functional HA activity of influenza vaccine. The vaccine stock was 10- and 50-fold diluted to make final concentration of 0.5 mg/ml in three different osmotic media, i.e. (A) iso-osmotic, (B) 300 mosM, and (C) 500 mosM, at pH 7.0 and 37°C. HA activity was measured after 2 h incubation at 37°C and plotted relative to that of control sample (vaccine in iso-osmotic medium at 4°C) before dilution. (Mean ± SD; n = 8). (TIF) [file pone.0066316.s005.tif]
